# Serum uric acid, gout, cardiometabolic diseases, and multimorbidity: a prospective cohort study using multi-state model

**DOI:** 10.1038/s41514-026-00413-6

**Published:** 2026-05-25

**Authors:** Chuanghai Wu, Guowei Zhong, Ying Yang, Baizhao Peng, Dexian Li, Zihao Jiang, Wen Fang, Shuai Ji, Xinghong Zhou, Hiu Yee Kwan, Yanyan Liu, Xiaoshan Zhao

**Affiliations:** 1 Nanfang Hospital, Southern Medical University, Guangzhou, Guangdong, China; 2 School of Traditional Chinese Medicine, Southern Medical University, Guangzhou, Guangdong, China; 3 Dongguan Hospital of Guangzhou University of Chinese Medicine, Dongguan, Guangdong, China; 4 School of Chinese Medicine, Hong Kong Baptist University, Hong Kong, China; 5 Guangdong Basic Research Center of Excellence for Integrated Traditional and Western Medicine for Qingzhi Diseases, Guangzhou, Guangdong, China; 6 The Seventh Affiliated Hospital of Southern Medical University, Foshan, Guangdong, China

**Keywords:** Biomarkers, Diseases, Endocrinology, Health care, Medical research, Risk factors

## Abstract

Multimorbidity, particularly cardiometabolic multimorbidity (CMM), is a growing global health challenge, defined by the co-occurrence of cardiometabolic diseases (CMDs) like type 2 diabetes, ischemic heart disease, and stroke. While serum uric acid (SUA) and gout have been linked to various chronic conditions, their roles in multimorbidity and the CMM trajectory remain unexplored in large populations. Using data from over 400,000 UK Biobank participants, we explored the associations between SUA, gout, 36 chronic conditions, and multimorbidity. A multi-state model was applied to investigate SUA and gout’s roles in the CMM trajectory, including transitions from CMD-free status to first CMD (FCMD), CMM, and death. Analyses were conducted for the overall population and stratified by sex. We observed that higher SUA levels and gout were associated with a higher likelihood of multimorbidity and multiple chronic conditions, particularly CMDs. Multi-state analysis revealed that both SUA and gout increased the risk of most transitions. Classifying FCMDs by specific CMDs further revealed distinct roles of SUA/gout in disease-specific transitions, even at the same stage. Sex-specific analyses showed a stronger impact in females compared to males. These findings highlight the importance of managing SUA levels and gout to prevent multimorbidity and slow CMM progression, particularly in females.

**Figure:**
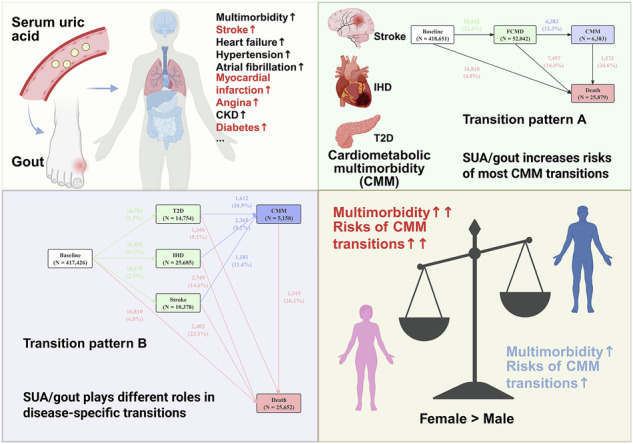

## Introduction

Multimorbidity, defined as the coexistence of two or more conditions in an individual, is increasingly prevalent, emerging as a growing global challenge^1^. Cardiometabolic multimorbidity (CMM), one of the most replicable multimorbidity profiles^2^, is characterized by the co-occurrence of at least two cardiometabolic diseases (CMDs), including type 2 diabetes (T2D), ischemic heart disease (IHD), and stroke^3^. CMM is associated with increased mortality, reduced life expectancy, greater functional decline, reduced quality of life, and rising healthcare costs^4–7^. Given the substantial burden of multimorbidity, particularly CMM, identifying potential modifiable risk factors is crucial.

Serum uric acid (SUA), the end product of purine metabolism, is essential for maintaining health within normal ranges. However, elevated SUA levels [i.e., hyperuricemia (HUA)] are recognized as the most important risk factor for gout, one of the most common inflammatory arthritis. Mendelian randomization studies have established a causal link between elevated SUA levels and an increased risk of gout^8,9^. Mechanistically, elevated SUA promotes the formation of monosodium urate crystals, which activate the NLRP3 inflammasome and trigger IL-1β–mediated inflammation, leading to the development of gout^10^. Both elevated SUA and gout are associated with an increased risk of various chronic conditions^8,11^, potentially through mechanisms such as systemic inflammation, oxidative stress, renin–angiotensin–aldosterone system activation, and endothelial dysfunction^11–13^. Despite this, most studies primarily focused on a limited number of chronic diseases, leaving the comprehensive relationship between SUA, gout, multiple chronic conditions, and multimorbidity in large populations insufficiently explored. Only two observational studies have examined the impact of SUA on the CMM development. One study identified an SUA-derived indicator associated with an increased risk of incident CMM in participants free of CMDs, regardless of the intermittent progression of a single CMD^14^. Another study assessed the effects of SUA changes on a single CMD and its progression to CMM in a limited population^15^. However, these analyses were fragmented, and the roles of SUA and gout in the complete CMM trajectory (i.e., from free of CMD to CMD, CMM, and finally death) were largely unknown^3^. Furthermore, sex is a crucial factor in multimorbidity clusters and their trajectories^1^. Although previous studies have identified sex-specific effects of SUA and gout on CMDs^16–18^, whether this phenomenon persists in the longitudinal progression of CMM remains unclear.

Therefore, we aimed to explore the associations between SUA, gout, 36 chronic conditions, and multimorbidity in the UK Biobank of 0.4 million participants. Based on these results, we further employed a multi-state model to investigate the roles of SUA and gout in the CMM trajectory, including transitions from CMD-free status to first cardiometabolic disease (FCMD), CMM, and finally death. Additionally, we examined the different roles of SUA and gout in all possible transitions by classifying FCMDs into specific conditions (i.e., T2D, IHD, and stroke). Notably, all analyses were conducted across the overall population, as well as separately for females and males, to comprehensively assess both general and sex-specific effects.

## Results

### SUA levels, 36 chronic conditions, and multimorbidity

Table 1 presents the baseline characteristics of the participants in the cross-sectional study. There were 362,833 participants without multimorbidity, and 106,096 with multimorbidity. Those with multimorbidity were more likely to be female and older. They were previous or current smokers, belonged to the most deprived group, had lower educational attainment, and had less drinking frequency. They also had higher SUA levels and body mass index (BMI), and a greater prevalence of gout at enrollment. Baseline characteristics stratified by sex are also presented in Table S1.

**Table 1:** Baseline characteristics by multimorbidity status at recruitment (*N* = 468,929)

Baseline characteristics	Overall	Without multimorbidity	With multimorbidity
Number	468,929	362,833	106,096
Male, *N* (%)	214,685 (45.8)	167,678 (46.2)	47,007 (44.3)
Age, mean (SD), years	56.53 (8.09)	55.75 (8.12)	59.20 (7.39)
Ethnicity, *N* (%)			
White British	413,981 (88.3)	320,001 (88.2)	93,980 (88.6)
Non-white British	52,742 (11.2)	41,175 (11.3)	11,567 (10.9)
Missing	2206 (0.5)	1657 (0.5)	549 (0.5)
Townsend deprivation index, *N* (%)			
1st quintile (least deprived)	93,868 (20.0)	75,053 (20.7)	18,815 (17.7)
2nd quintile	93,819 (20.0)	74,141 (20.4)	19,678 (18.5)
3th quintile	93,682 (20.0)	73,455 (20.2)	20,227 (19.1)
4th quintile	93,776 (20.0)	72,186 (19.9)	21,590 (20.3)
5th quintile (most deprived)	93,784 (20.0)	67,998 (18.7)	25,786 (24.3)
Drinking frequency, *N* (%)			
Frequent	324,326 (69.2)	259,570 (71.5)	64,756 (61.0)
Infrequent	105,944 (22.6)	77,499 (21.4)	28,445 (26.8)
Never	37,625 (8.0)	24,997 (6.9)	12,628 (11.9)
Missing	1034 (0.2)	767 (0.2)	267 (0.3)
Smoking status, *N* (%)			
Never	255,323 (54.4)	204,034 (56.2)	51,289 (48.3)
Previous	162,006 (34.5)	119,280 (32.9)	42,726 (40.3)
Current	49,221 (10.5)	37,815 (10.4)	11,406 (10.8)
Missing	2379 (0.5)	1704 (0.5)	675 (0.6)
Education, *N* (%)			
College or above	151,865 (32.4)	123,888 (34.1)	27,977 (26.4)
High school	231,506 (49.4)	180,511 (49.8)	50,995 (48.1)
Less than high school	79,964 (17.1)	54,395 (15.0)	25,569 (24.1)
Missing	5594 (1.2)	4039 (1.1)	1555 (1.5)
Body mass index, *N* (%)			
<25.0 Kg/m^2^	154,541 (33.0)	129,880 (35.8)	24,661 (23.2)
25–29.9 Kg/m^2^	200,628 (42.8)	157,722 (43.5)	42,906 (40.4)
≥30.0 Kg/m^2^	113,760 (24.3)	75,231 (20.7)	38,529 (36.3)
Urate, mean (SD), μmmol/L	309.21 (80.43)	305.59 (78.44)	321.59 (85.77)
Gout at baseline, *N* (%)	9945 (2.1)	6138 (1.7)	3807 (3.6)

Categorical data presented as number (percentages) and continuous data presented as mean (standard deviation).

*SD* standard deviation.

**Fig. 1:**
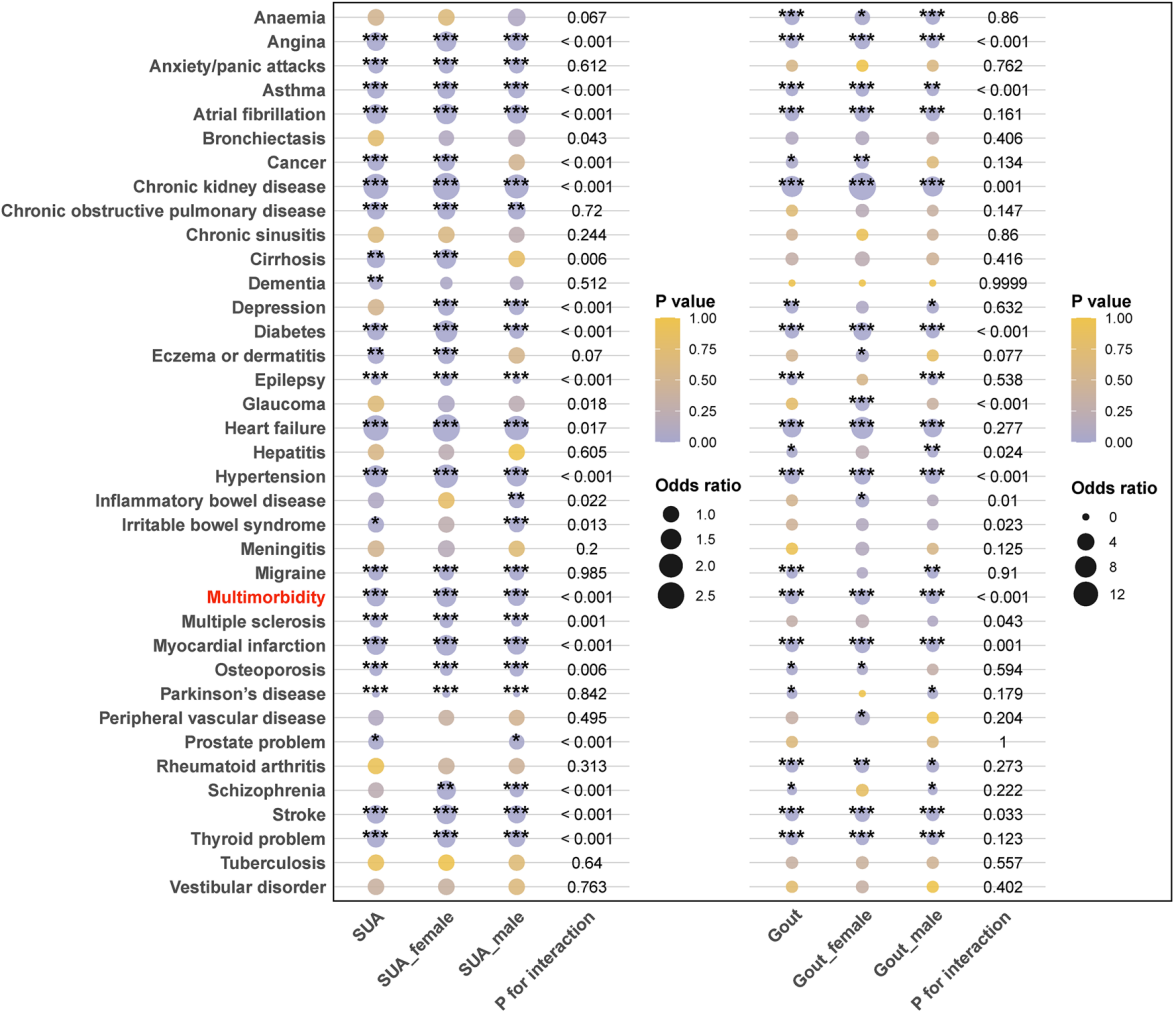
Associations between SUA, gout, multimorbidity, and 36 chronic conditions (*N* = 468,929). The heatmap displays ORs for multimorbidity and 36 chronic conditions associated with each SD increase in SUA levels (left panel) and gout (right panel). The model was adjusted for sex (male/female), age at recruitment (continuous), ethnic background (white British/non-white British), and the Townsend deprivation index (1st/2nd/3rd/4th/5th quintile). For sex-specific analyses, sex was not included in the model. Each panel, from left to right, presents results for the overall population, females, males, and interaction significance. Bubble color indicates the P value, while bubble size reflects the magnitude of ORs, with larger bubbles representing larger ORs. ***P < 0.001, **P < 0.01, *P < 0.05. CI confidence interval, OR odds ratios, SD standard deviation, SUA serum uric acid.

As shown in Fig. 1, Fig. S1, and Table S2, per standard deviation (SD) increase in SUA was associated with a 26.7% higher likelihood of multimorbidity [odds ratio (OR): 1.267, 95% confidence interval (CI): 1.256–1.277] and a 14% increase in the number of chronic conditions [relative risk (RR): 1.142, 95% CI: 1.138–1.146]. SUA was significantly associated with 23 conditions, 14 of which were positively correlated, such as heart failure, chronic kidney disease (CKD), hypertension, atrial fibrillation, myocardial infarction (MI), angina, stroke, cirrhosis, thyroid disorders, chronic obstructive pulmonary disease, and diabetes. Conversely, SUA exhibited negative associations with 9 conditions, such as Parkinson’s disease, epilepsy, multiple sclerosis, osteoporosis, and dementia.

Furthermore, sex differences were observed (Fig. 1, Fig. S2). The association between SUA and multimorbidity was stronger in females (OR: 1.406, 95% CI: 1.390–1.422) than in males (OR: 1.151, 95% CI: 1.138–1.165) (*P*_interaction_ < 0.001). SUA was linked to more chronic conditions in females compared to males (*P*_interaction_ < 0.001) (Table S2). Notably, females were more susceptible to the detrimental effects of SUA in certain conditions, such as heart failure, CKD, diabetes, hypertension, MI, atrial fibrillation, angina, and stroke.

### Gout, 36 chronic conditions, and multimorbidity

Among the 9945 participants with gout at baseline, 3807 had prevalent multimorbidity (Table 1). Compared to participants without gout, those with gout had 94.6% higher odds of multimorbidity (OR: 1.946, 95% CI: 1.865–2.031) and a greater number of chronic conditions (RR: 1.431, 95% CI: 1.405–1.457) (Fig. 1, Fig. S3, Table S2). Gout showed significant associations with 20 conditions, 15 of which were positively correlated, such as CKD, heart failure, hypertension, anemia, atrial fibrillation, diabetes, stroke, angina, and MI. In contrast, gout was inversely associated with 6 conditions, including schizophrenia, Parkinson’s disease, hepatitis, epilepsy, migraine, and osteoporosis.

As Fig. 1 and Fig. S4 indicate, the association between gout and multimorbidity was more pronounced in females (OR: 2.758, 95% CI: 2.433–3.125) than in males (OR: 1.843, 95% CI: 1.761–1.929) (*P*_interaction_ < 0.001). Gout was linked to more chronic conditions in females compared to males (*P*_interaction_ < 0.001) (Table S2). Females exhibited a higher susceptibility to gout’s adverse effects in seven conditions, including CKD, diabetes, hypertension, MI, angina, stroke, and asthma (*P*_interaction_ < 0.05).

### SUA levels, gout, and the CMM trajectory (transition pattern A)

**Fig. 2:**
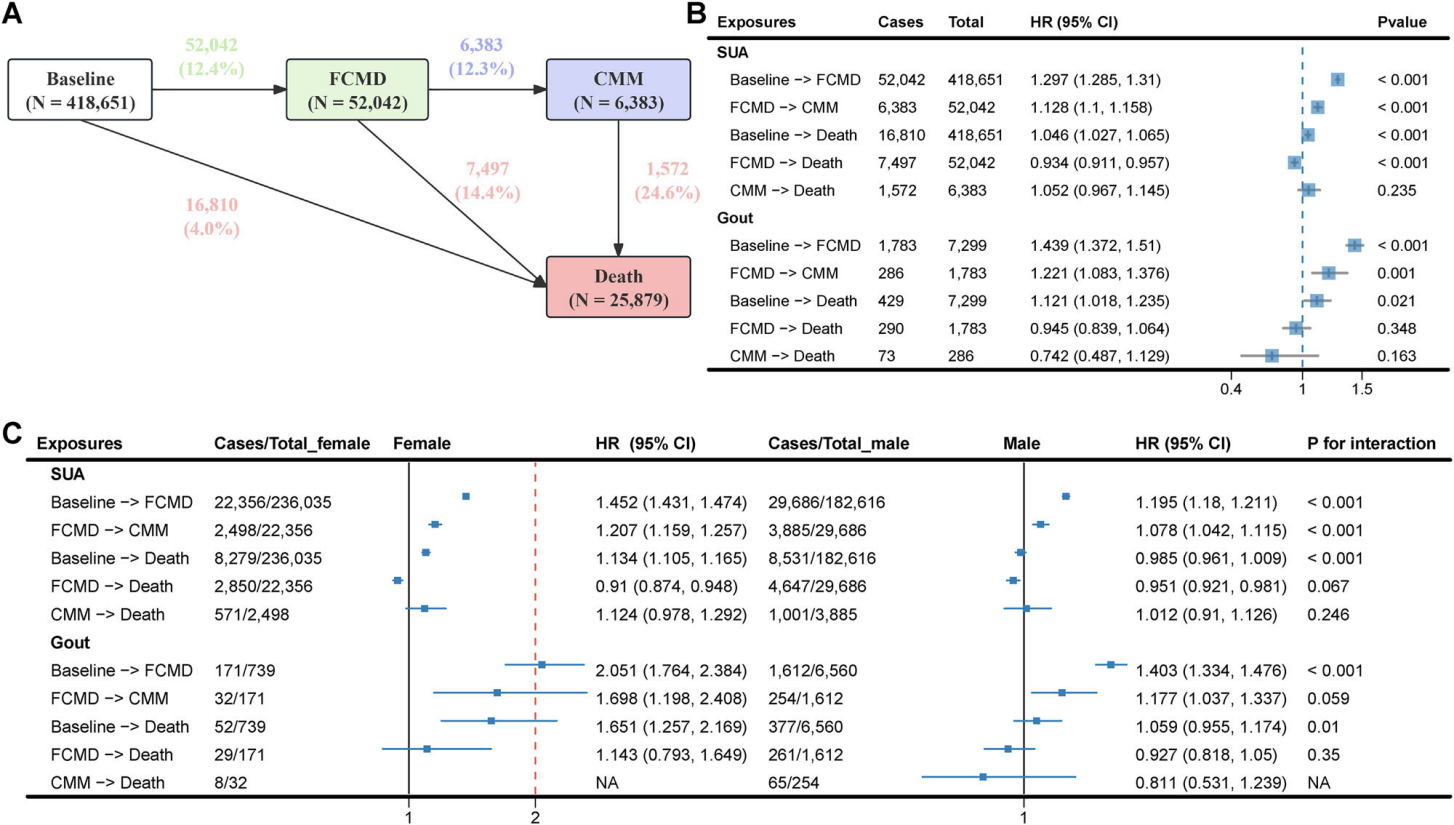
Associations between SUA, gout, and the trajectory of CMM (transition pattern A) (*N* = 418,651). **A** Numbers (percentages) of participants in transition pattern A from baseline to FCMD, CMM, and death. FCMD was defined as the first occurrence of any of the following three CMDs (type 2 diabetes, ischemic heart disease, and stroke). CMM was defined as the co-occurrence of at least two of the CMDs. HRs (95% CIs) for SUA and gout in the overall population (**B**) and by sex (**C**) for transition pattern A. For SUA analyses, associations are reported per standard deviation increase in SUA levels. The model was adjusted for sex (male/female), age at recruitment (continuous), ethnic background (white British/non-white British), and the Townsend deprivation index (1st/2nd/3rd/4th/5th quintile). For sex-specific analyses, sex was not included in the model. CI confidence interval, CMD cardiometabolic diseases, CMM cardiometabolic multimorbidity, FCMD first cardiometabolic disease, HR hazard ratio, NA not available, SUA serum uric acid.

Our cross-sectional analysis demonstrated that both SUA and gout significantly increased the odds of three CMDs—diabetes, IHD (MI, angina), and stroke—overall and by sex. Building on these findings, our cohort study further investigates CMDs and CMM. Table 2 presents the baseline characteristics of participants in the cohort study. Compared to healthy individuals (i.e., CMD-free), those who progressed into CMM (i.e., CMM survivors and dead with CMM) were more likely to be male, older, previous or current smokers, belong to the most deprived group, have lower educational attainment, and report infrequent alcohol consumption. They had higher SUA levels, BMI, and a greater prevalence of gout at enrollment. The baseline characteristics stratified by sex are also presented in Tables S3–4. As shown in Fig. 2A, over a 12.7-year median follow-up period (interquartile range: 11.8, 13.5), 52,042 (12.4%) participants developed at least one CMD, with 6383 (12.3%) subsequently developing CMM. During the follow-up, 25,879 participants died, including 16,810 without CMD, 7497 with FCMD, and 1572 after developing CMM.

**Table 2:** Baseline characteristics by incident disease status during follow-up (*N* = 418,651)

	CMDfree	FCMD survivor	CMMsurvivor	Dead without CMD	Dead with FCMD	Dead with CMM
Number	349,799	38,162	4811	16,810	7497	1572
Male, *N* (%)	144,399 (41.3)	21,154 (55.4)	2884 (59.9)	8531 (50.7)	4647 (62.0)	1001 (63.7)
Age, mean (SD), years	55.29 (8.07)	58.88 (7.35)	60.22 (6.90)	60.74 (6.78)	61.86 (6.31)	62.77 (5.79)
Ethnicity, *N* (%)						
White British	309,677 (88.5)	33,426 (87.6)	4100 (85.2)	15,412 (91.7)	6795 (90.6)	1375 (87.5)
Non-white British	38,649 (11.0)	4518 (11.8)	672 (14.0)	1316 (7.8)	665 (8.9)	183 (11.6)
Missing	1473 (0.4)	218 (0.6)	39 (0.8)	82 (0.5)	37 (0.5)	14 (0.9)
Townsend deprivation index, *N* (%)						
1st quintile (least deprived)	73,278 (20.9)	7202 (18.9)	767 (15.9)	3243 (19.3)	1284 (17.1)	246 (15.6)
2nd quintile	72,020 (20.6)	7337 (19.2)	840 (17.5)	3315 (19.7)	1292 (17.2)	261 (16.6)
3th quintile	71,002 (20.3)	7503 (19.7)	925 (19.2)	3213 (19.1)	1434 (19.1)	287 (18.3)
4th quintile	69,713 (19.9)	7681 (20.1)	933 (19.4)	3307 (19.7)	1532 (20.4)	334 (21.2)
5th quintile (most deprived)	63,786 (18.2)	8439 (22.1)	1346 (28.0)	3732 (22.2)	1955 (26.1)	444 (28.2)
Drinking frequency, *N* (%)						
Frequent	248,891 (71.2)	25,123 (65.8)	2877 (59.8)	11,561 (68.8)	5038 (67.2)	1001 (63.7)
Infrequent	76,354 (21.8)	9275 (24.3)	1299 (27.0)	3691 (22.0)	1638 (21.8)	362 (23.0)
Never	23,920 (6.8)	3645 (9.6)	621 (12.9)	1521 (9.0)	793 (10.6)	200 (12.7)
Missing	634 (0.2)	119 (0.3)	14 (0.3)	37 (0.2)	28 (0.4)	9 (0.6)
Smoking status, *N* (%)						
Never	202,304 (57.8)	18,885 (49.5)	2161 (44.9)	7245 (43.1)	2932 (39.1)	538 (34.2)
Previous	113,428 (32.4)	14,183 (37.2)	1856 (38.6)	6479 (38.5)	2907 (38.8)	661 (42.0)
Current	32,630 (9.3)	4859 (12.7)	760 (15.8)	2966 (17.6)	1590 (21.2)	352 (22.4)
Missing	1437 (0.4)	235 (0.6)	34 (0.7)	120 (0.7)	68 (0.9)	21 (1.3)
Education, *N* (%)						
College or above	123,112 (35.2)	9969 (26.1)	1038 (21.6)	4495 (26.7)	1760 (23.5)	307 (19.5)
High school	175,173 (50.1)	18,984 (49.7)	2231 (46.4)	7858 (46.7)	3317 (44.2)	642 (40.8)
Less than high school	47,994 (13.7)	8609 (22.6)	1445 (30.0)	4234 (25.2)	2285 (30.5)	590 (37.5)
Missing	3520 (1.0)	600 (1.6)	97 (2.0)	223 (1.3)	135 (1.8)	33 (2.1)
Body mass index, *N* (%)						
<25.0 Kg/m^2^	129,252 (37.0)	8390 (22.0)	761 (15.8)	5682 (33.8)	2035 (27.1)	319 (20.3)
25–29.9 Kg/m^2^	150,424 (43.0)	16,660 (43.7)	2002 (41.6)	7188 (42.8)	3272 (43.6)	618 (39.3)
≥30.0 Kg/m^2^	70,123 (20.0)	13,112 (34.4)	2048 (42.6)	3940 (23.4)	2190 (29.2)	635 (40.4)
Urate, mean (SD), μmmol/L	300.99 (77.48)	332.16 (80.94)	344.64 (82.10)	316.91 (81.38)	334.24 (85.31)	353.24 (89.03)
Gout at baseline, *N* (%)	5087 (1.5)	1207 (3.2)	213 (4.4)	429 (2.6)	290 (3.9)	73 (4.6)

Categorical data presented as number (percentages) and continuous data presented as mean (standard deviation).

*CMD* cardiometabolic disease, *CMM* cardiometabolic multimorbidity, *FCMD* first cardiometabolic disease, *SD* standard deviation.

As indicated by the traditional Cox models, both SUA and gout significantly increased the risk of FCMD, CMM, and all-cause mortality, particularly in females (Table S5). As shown in Fig. 2A, B, the multi-state analyses further revealed different impacts of SUA/gout on the temporal trajectory of CMM. Both SUA and gout significantly increased the risk of the transition from healthy to FCMD and subsequent CMM, with hazard ratios (HRs) (95% CIs) of 1.297 (1.285–1.31) and 1.128 (1.1–1.158) for SUA, as well as 1.439 (1.372–1.510) and 1.221 (1.083–1.376) for gout. Besides, both SUA (HR: 1.046, 95% CI: 1.027–1.065) and gout (HR: 1.121, 95% CI: 1.018–1.235) elevated risks of the transition from baseline to death. However, for the transition from FCMD to death, opposite effects were observed for SUA (HR: 0.934, 95% CI: 0.911–0.957) and gout (HR: 0.945, 95% CI: 0.839–1.064). Furthermore, no significant associations were found between SUA or gout and the transition from CMM to death.

When stratified by sex, the results for SUA and gout aligned with the overall trend, but significant sex differences were noted in various transitions (Fig. 2C). Specifically, females showed greater susceptibility to SUA than males in transitions from baseline to FCMD, FCMD to CMM, and baseline to death (all *P*_interaction_ < 0.001). Similar patterns were noted for gout, with stronger effects in females for these transitions (*P*_interaction_ < 0.05), except for FCMD to CMM (*P*_interaction_ = 0.059). No significant sex differences were found in the effects of SUA and gout on the transition from morbidity to mortality.

### SUA levels, gout, and the CMM trajectory (transition pattern B)

**Fig. 3:**
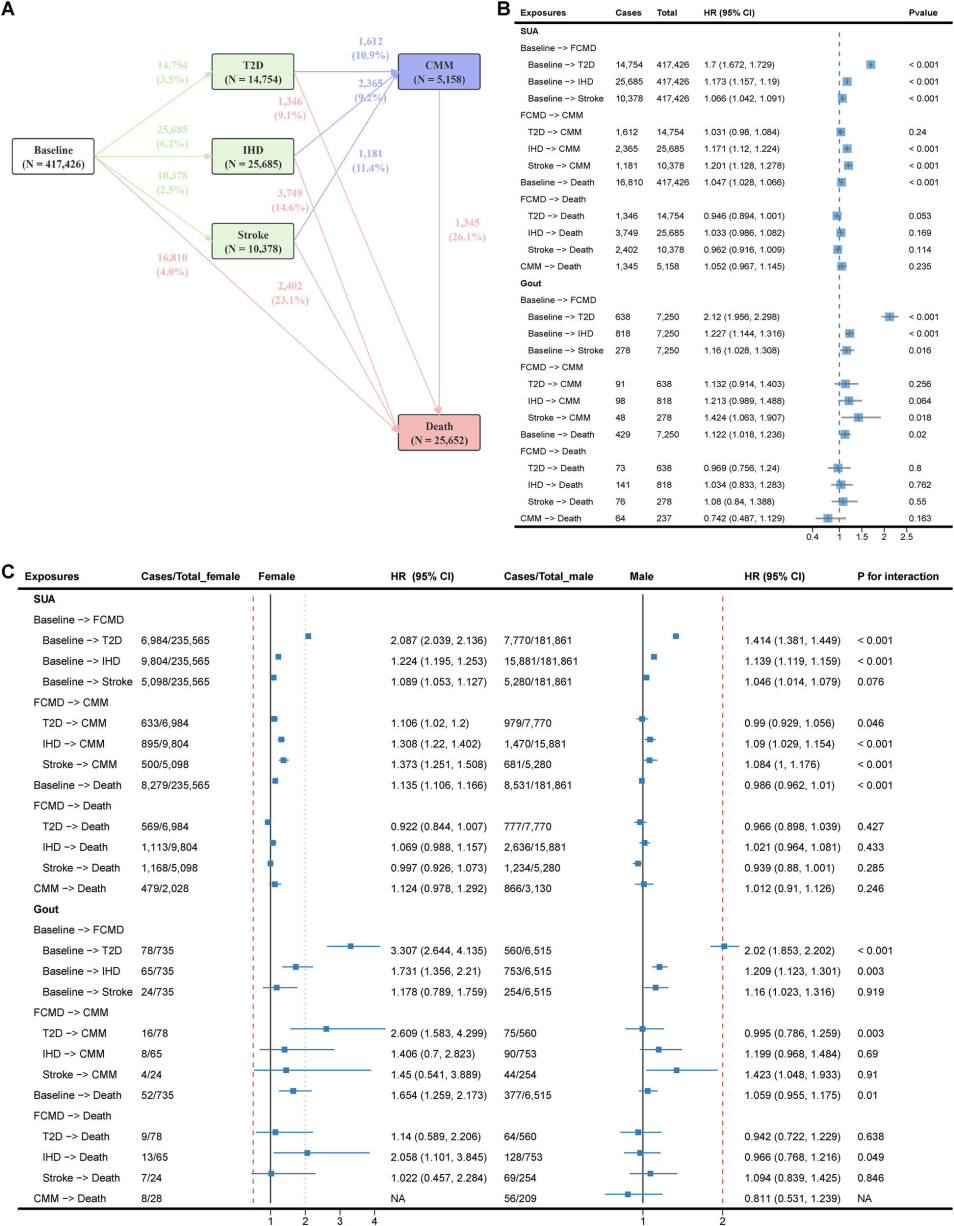
Associations between SUA, gout, and the trajectory of CMM (transition pattern B) (*N* = 417,426). **A** Numbers (percentages) of participants in transition pattern B from baseline to one of CMDs (type 2 diabetes, ischemic heart disease, and stroke), then to CMM, and finally death. CMM was defined as the co-occurrence of at least two of the CMDs. HRs (95% CIs) for SUA and gout in the overall population (**B**) and by sex (**C**) for transition pattern B. For SUA analyses, associations are reported per standard deviation increase in SUA levels. The model was adjusted for sex (male/female), age at recruitment (continuous), ethnic background (white British/non-white British), and the Townsend deprivation index (1st/2nd/3rd/4th/5th quintile). For sex-specific analyses, sex was not included in the model. CI confidence interval, CMD cardiometabolic diseases, CMM cardiometabolic multimorbidity, FCMD first cardiometabolic disease, HR hazard ratio, IHD ischemic heart disease, NA not available, SUA serum uric acid, T2D type 2 diabetes.

When FCMDs were further divided into T2D, IHD, and stroke, 14,754 (3.5%) participants had T2D, 25,685 (6.5%) had IHD, and 10,378 (2.5%) had stroke. Of these, 1612 (10.9%), 2365 (9.2%), and 1181 (11.4%) developed CMM, respectively (Fig. 3A). Both SUA and gout were linked to an increased risk of transition from baseline to each CMD, with the strongest association observed for T2D [HR (95% CI): 1.7 (1.672–1.729) for SUA; 2.12 (1.956–2.298) for gout], followed by IHD [1.173 (1.157–1.19) for SUA; 1.227 (1.144–1.316) for gout], and stroke [1.066 (1.042–1.091) for SUA; 1.16 (1.028–1.308) for gout]. For CMM following FCMD, SUA increased the risk in individuals with IHD and stroke but not T2D, while gout was significantly associated only with stroke progression. In mortality analyses, both SUA and gout elevated the risk of transition from baseline to death, but not from FCMDs or CMM to death (Fig. 3B).

Sex-specific effects of SUA and gout were observed in transition pattern B (Fig. 3C). The influence of SUA and gout on transitions from baseline to T2D and IHD was more pronounced in females (*P*_interaction_ < 0.05), but no such difference was found for stroke. Moreover, females were more susceptible to SUA in transitions from all three FCMDs to CMM, and to gout in the transition from T2D to CMM (*P*_interaction_ < 0.05). SUA and gout also had a stronger impact on the transition from baseline to death in females. Except for gout’s effect on the transition from IHD to death (*P*_interaction_ = 0.049), no sex-specific associations were observed for other transitions from FCMDs or CMM to mortality.

### Subgroup and sensitivity analyses

In addition to sex, we stratified the results by age, ethnic background, and the Townsend deprivation index. Age-based stratification revealed stronger associations between SUA, gout, and multimorbidity in individuals under 60 years old compared to those above 60 (*P*_interaction_ < 0.001 for SUA; *P*_interaction_ = 0.0327 for gout). No significant interactions were found in other subgroups (Table S6).

Sensitivity analyses confirmed that the cross-sectional associations between SUA, gout, and multimorbidity, overall and by sex, remained robust (Fig. S5, Tables S7–8). The overall and sex-specific effects of SUA and gout on transition pattern A were largely unchanged, except for the relationship between gout and the risk of transitioning from baseline to death after further adjustment for SUA and HUA (Tables S9–10). Similarly, the results for transition pattern B remained robust (Fig. S6).

## Discussion

In this large prospective cohort study of 0.4 million adults, we examined the effects of SUA and gout on 36 chronic conditions, multimorbidity, and the CMM trajectory. Cross-sectional analyses suggested that both higher SUA levels and gout were associated with an increased likelihood of multimorbidity and multiple chronic conditions. Multi-state analysis revealed that higher SUA levels and gout were significantly linked to higher risks of most transitions (e.g., from baseline to FCMD, from FCMD to CMM, and from baseline to death). When FCMDs were divided into three specific conditions, the effects of SUA and gout varied by condition within the same transition stage. Notably, the strongest effects were observed for T2D in the transition from baseline to FCMD and for stroke in the transition from FCMD to CMM. Sex-stratified analyses showed that both females and males exhibited similar trends to the overall population, with SUA and gout having a more pronounced impact in females.

A recent cross-sectional study in China, defining multimorbidity as the presence of 15 chronic conditions, found that individuals with elevated SUA levels (>430 μmol/L) are more likely to experience multimorbidity (OR: 1.36, 95% CI: 1.22–1.51) compared to those within the normal SUA range (120–430 μmol/L)^19^. In our study, we assessed multimorbidity across 36 chronic conditions in a cohort of 400,000 individuals from the UK. Using the effect size of HUA for comparison, we found a similar yet stronger impact in our cohort (OR: 1.723, 95% CI: 1.691–1.757) (Table S7). With a broader range of well-documented conditions and a larger sample size, our study substantially reinforces the association between SUA and multimorbidity. Another recent study based on the China Kadoorie Biobank (CKB) finds that gout patients have a higher prevalence of ≥2 co-morbidities (OR: 4.11, 95% CI: 3.56–4.74)^11^. Our study also revealed a significant, though smaller, adverse effect of gout (OR: 1.946, 95% CI: 1.865–2.031). This discrepancy may stem from differences in gout definitions across studies. In the CKB cohort, nearly all gout cases were hospitalized, indicating more severe disease, whereas our cohort included diagnoses across primary care, hospital admissions, and mortality records. Consequently, our effect size more accurately reflects the general severity of gout, while minimizing potential selection bias. Additionally, we employed a quasi-Poisson mixed-effects model to assess the relationships between SUA, gout, and the number of chronic conditions, a methodology not previously reported (Table S2). This reinforces the negative impact of SUA and gout on multimorbidity.

Only one observational study has simultaneously examined the effects of SUA and gout on comorbidities using the UK Biobank^20^. This case-control study identifies significant associations with six comorbidities: CKD, hypertension, diabetes, IHD, hyperlipidemia, and congestive heart failure, and highlights an SUA-independent relationship between gout and these conditions. Our study extends these findings by examining 36 chronic conditions. We replicated the associations with IHD, hypertension, CKD, and heart failure, confirming that gout’s impact remains independent of SUA (Fig. 1, Table S8). Additionally, we reinforce the associations between SUA, gout, and a wider range of conditions, particularly two CMDs (stroke and diabetes), atrial fibrillation, and Parkinson’s disease, consistent with findings in other populations^8,11,21^. Our study observed that both SUA and gout were significantly associated with a protective effect on prevalent osteoporosis, which may be explained by the antioxidant properties of SUA^22,23^. However, our findings differ from two recent studies, which reported that higher SUA levels and gout are associated with an increased risk of osteoporosis^23,24^. Moreover, the effects of SUA and gout on conditions such as asthma, epilepsy, and migraine have not been reported in previous studies. Given these controversial and underexplored findings, well-designed, large-sample longitudinal studies and Mendelian randomization analyses are warranted to further validate these associations.

CMM is one of the most replicable multimorbidity profiles and has attracted growing interest from researchers recently^2^. The adverse impacts of SUA and gout on incident CMDs are well-documented^11,25,26^. A study of Chinese adults found that SUA changes (i.e., keeping or rising to HUA) are associated with increased odds of cardiovascular diseases following diabetes (OR:1.67, 95% CI: 1.15–2.43)^15^. Consistent with these findings, our traditional Cox regression analyses also identified associations between SUA, gout, and increased risks of incident FCMD and CMM (Table S5). However, these analytical strategies have limitations. Focusing on a single stage of the CMM trajectory (e.g., CMD-free to CMM) may regard other outcomes (e.g., CMD-free to FCMD) as censored, overlooking the varying impacts of SUA and gout across different stages^3^. Moreover, given the impact of SUA and gout on mortality^8,27^, individuals who died during follow-up may have had a higher risk of developing FCMD or CMM. Thus, studies that focus solely on FCMD or CMM may neglect the competing risk of mortality, potentially distorting the effects of SUA and gout on the CMM trajectory.

To address these limitations, we employed a multi-state model that allows us to assess the effects of SUA and gout across all possible transitions in the CMM trajectory, while accounting for competing risks^3^. In the transition pattern A analysis, we found that both SUA and gout increased the risk of transitions from healthy to FCMD and subsequently to CMM (Fig. 2A–C). Additionally, SUA and gout negatively influenced the transition from baseline to death, consistent with prior studies^8,27^. However, no similar adverse effects of SUA and gout on mortality risk following FCMD or CMM were observed in our study. These findings partially align with previous studies that find no significant association between SUA and the prognosis (e.g., death) in stroke patients^28,29^. Similarly, another cohort study supports our results, suggesting that gout does not influence the risk of death in T2D patients^30^. In contrast, a study in the US population showed that each 1 mg/mL increment in SUA increases the mortality risk in T2D patients (HR: 1.07, 95% CI: 1.02–1.12)^31^. This discrepancy may be attributed to differences in population, sample size, and analytical methods. Further studies using multi-state models are warranted to validate these findings across diverse and large populations. In conclusion, higher SUA levels and gout are independent risk factors for CMM progression. Incorporating SUA and gout into predictive models could enhance the accuracy of forecasting CMM development and aid clinical decision-making. Early interventions to maintain SUA within physiological levels and manage gout may slow CMM progression, thereby reducing healthcare costs and economic burden.

In the analyses of transition pattern B, SUA/gout exhibited differential effects across disease-specific transitions (Fig. 3A–C). The most substantial impact was observed on T2D for the transition from baseline to FCMD. These findings partially align with a previous CKB study, which reported that the effects of SUA and gout on T2D are slightly more pronounced than on IHD and stroke^11^. Despite this, SUA and gout’s effects on CMM following T2D appeared minimal. Previous research also found that neither SUA nor gout increases cardiovascular disease risk in individuals with T2D^30,32^. Additionally, our study revealed the strongest effect of SUA and gout in stroke patients for the transition from FCMD to CMM. Consistent with this, a prior study from New Zealand found that gout is linked to an increased likelihood of T2D among stroke patients^33^. This may be explained by chronic systemic inflammation following stroke, which heightens the risk of stroke-related comorbidities, including diabetes^34^. Given the interaction between SUA/gout and systemic inflammation in diabetes development^35^, it is plausible that the combination of SUA/gout and post-stroke inflammation contributes to the progression to CMM. Overall, our study highlights disease-specific transitions and underscores the potential for developing targeted prevention strategies for CMM, particularly by managing SUA levels and preventing gout in individuals at risk for T2D and stroke.

Sex-specific effects in our study largely mirror the overall population trend, with adverse outcomes more pronounced in females. These include a higher burden of chronic conditions, greater multimorbidity prevalence, and more rapid progression of CMM. With 0.4 million participants, our study further strengthens previous evidence that females are more vulnerable to the adverse effects of SUA/gout on various comorbidities^36^, CMDs^16–18^, and mortality^37,38^. Leveraging long follow-up periods and a multi-state model, we present the first comprehensive analysis of sex-specific impacts across each independent transition in the CMM trajectory, a novel contribution. One potential mechanism may be attributed to inflammation, which has been demonstrated to play a pivotal role in the development of CMM^39,40^. Inflammation itself exhibits significant sexual dimorphism, which may arise from both sex chromosome complement (i.e., XY or XX) and sex steroids (e.g., testosterone and estrogens)^41^. We speculate that elevated inflammation levels, mediated by X chromosome-linked genes and sex hormones, may contribute to the increased susceptibility of females to SUA- and gout-associated CMM risk. Our findings highlight the urgent need for targeted interventions and tailored preventive strategies for SUA and gout in females, who are often underrepresented in clinical research. Addressing this gap is essential for reducing gender inequality in healthcare, alleviating disease burden, and improving health outcomes.

Our study has several strengths. The primary advantage is the use of a multi-state model, which allows us to comprehensively assess the differential effects of SUA and gout within the CMM trajectory, while mitigating the confounding influence of competing risks from death. Leveraging the large UK Biobank sample facilitates the analysis of disease- and sex-specific associations. Additionally, we investigate the effects of SUA and gout across 36 chronic conditions, providing broader insights into their roles. However, several limitations should be considered. Firstly, mean imputation and the missing-indicator method were used to handle missing data, which may introduce bias and potentially affect the results. More sophisticated approaches, such as multiple imputation, are recommended for future studies to address missing values more robustly. Secondly, although our study was conducted in a large cohort, gout is less prevalent in females than in males^42^. Additionally, incident CMM cases in females during follow-up were limited, which may have reduced statistical power for certain transitions, such as from CMM to death. These findings should be validated in a larger cohort with a sufficient number of female gout cases. Thirdly, in the analysis of transition pattern B, we excluded individuals diagnosed with two or more CMDs on the same day, as done in previous studies^3,43^. However, this approach may introduce selection bias and potentially affect our results. In the sensitivity analysis, when we randomly selected one CMD to represent the earliest diagnosis for that day and re-analyzed the data, the results were consistent with those of the main analysis. Therefore, we consider these findings to be relatively robust. Furthermore, as an observational study, causal relationships cannot be inferred, necessitating further research, such as well-designed Mendelian randomization studies, to establish causality. Finally, although racial and ethnic disparities in multimorbidity are previously documented, our study predominantly involved individuals of white British descent^1,44^, limiting the diversity of the sample and potentially affecting the generalizability of our findings.

In conclusion, our study reveals a significant association between higher SUA levels, gout, and an increased risk of multiple chronic conditions, multimorbidity, and CMM progression, particularly in females. These findings underscore the importance of managing SUA levels and gout in clinical practice to prevent multimorbidity and slow CMM progression. Further research is needed to elucidate the underlying mechanisms and develop targeted interventions to improve patient outcomes across various CMM stages, especially in females.

## Methods

### Study design and participants

The UK Biobank is a large-scale, nationwide prospective cohort study that enrolled over 500,000 individuals aged 37 to 73 years from across the UK between 2006 and 2010. Data on sociodemographics, lifestyle, and health were collected through various methods, such as touchscreen questionnaires, interviews, and physical measurements. Biological samples (blood, urine, and saliva) were also collected. The study was approved by the North West Multicenter Research Ethics Committee (REC reference 21:/NW/0157), and written informed consent was provided by all participants. The UK Biobank has been conducted in accordance with ethical standards, the Declaration of Helsinki, and relevant national and international guidelines. Detailed information on the UK Biobank can be found in a prior publication^45^. Our research was conducted under project number 90369.

This study used cross-sectional and cohort analyses to examine the roles of SUA and gout. As shown in Fig. S7, the initial cohort comprised 502,402 adults aged 37–73. After excluding 33,473 participants missing SUA data, 468,929 individuals remained for the cross-sectional analysis of multimorbidity and 36 chronic conditions. Subsequently, 50,278 participants with baseline diagnoses of T2D (23,992), IHD (25,246), or stroke (8210) were excluded, leaving 418,651 individuals for the cohort analysis of CMM trajectory.

### Exposures

SUA levels (field ID 30880) were measured using uricase PAP analysis on a Beckman Coulter AU5800. These SUA values were subsequently standardized to SD for further analysis. Baseline gout (ICD code M10) was defined based on the “first occurrence” (category ID 1712, field ID 131859 for gout), which integrated data from primary care records, hospital admissions, death registers, and self-reported medical conditions^46^ (Table S11).

### Outcomes

The UK Biobank collected self-reported medical data (field ID 20002), with diagnoses validated by healthcare professionals. In the cross-sectional analysis, 36 chronic conditions were included, and multimorbidity was defined as the presence of ≥2 conditions, as per previous research^47^ (Table S12). For the cohort study, FCMD was defined as the first occurrence of any of three CMDs—T2D, IHD, or stroke—while CMM was defined as the co-occurrence of ≥2 CMDs, consistent with previous studies. Specifically, IHD and stroke were defined using ICD codes I20-I25 and I60-I69, respectively, while T2D was categorized under both E11 and E14, as supported by previous studies^3,43^. Additionally, incident cases of all-cause death were ascertained through linkage to national death registries (Table S11).

### Covariates

We considered sex (male/female), age at recruitment (continuous), ethnic background (white British/non-white British), and the Townsend deprivation index (1st/2nd/3rd/4th/5th quintile) as potential covariates, based on prior knowledge^48^. Details of covariates used in the main and sensitivity analyses were provided in Table S11.

### Statistical analysis

Baseline characteristics were described according to the type and distribution of each variable. Continuous variables with normal distribution were presented as means and SD. Categorical variables were described as numbers and percentages. Both cross-sectional and cohort analyses were adjusted for sex, age at recruitment, ethnic background, and the Townsend deprivation index. Missing data on the covariates were handled using mean imputation for continuous variables and missing indicators for categorical variables. Logistic regression models were employed in cross-sectional analyses to calculate the ORs and 95% CIs for the association between SUA, gout, multimorbidity, and 36 chronic conditions. SUA was standardized for model inclusion, while gout status (with/without gout) was treated as a categorical variable, with “without gout” as the reference group (the same as below). Quasi-Poisson mixed-effects models were employed to estimate the RR and 95% CI for the associations between SUA, gout, and the number of 36 chronic conditions^49^. For the cohort study, follow-up durations were calculated from the enrollment date to the occurrence of outcome events, death, loss to follow-up, or censoring date (31 December 2021), whichever occurred first. Cox proportional hazards regression models were used to assess the HRs and 95% CIs, and the proportional hazards assumption was evaluated using Schoenfeld residuals.

Subsequently, a multi-state model was employed to assess the effects of SUA and gout on each transition phase of CMM progression and prognosis (i.e., from CMD-free to FCMD, CMM, and death). The multi-state model, an extension of the traditional Cox model, provides unique advantages by simultaneously evaluating certain factors’ effects on different phases of a process, while considering competing risks from death. Competing risks are naturally accounted for within the multi-state framework by explicitly defining all possible transitions between states (baseline, FCMD, CMM, and death). For each transition, a transition-specific hazard is estimated, with the risk set for a given transition—such as from CMD-free to FCMD—comprising only individuals currently in the origin state who have not yet experienced FCMD, CMM, or death^50,51^. Based on the natural history of CMM and previous studies^3,43^, we developed a transition pattern comprising five stages (transition pattern A): (1) baseline to FCMD, (2) FCMD to CMM, (3) baseline to death, (4) FCMD to death, and (5) CMM to death (Fig. 2A). To establish the chronological order of events, we recalculated event dates for individuals who entered different events on the same day. Specifically, the entering date for the theoretically prior state (e.g., CMM) was set to 0.5 days before the entering date of the latter state (e.g., death). To thoroughly assess the impact of SUA and gout on the progression from baseline to various FCMDs, CMM, and death, we further classified FCMDs into T2D, IHD, and stroke, and developed transition pattern B, which includes 11 stages (Fig. 3A). As we could not determine the theoretical prior state among the three FCMDs, we excluded participants who entered different FCMDs on the same day (1225), leaving 417,426 individuals for further analyses.

To comprehensively assess the overall and sex-specific effects of SUA and gout, all analyses in both the cross-sectional and cohort studies were stratified by the overall population, females, and males. Additionally, subgroup analyses in the cross-sectional study were further conducted based on age, ethnic background, and Townsend deprivation index. Interaction significance was evaluated using the likelihood ratio test^52^.

Several sensitivity analyses were conducted to assess the robustness of the results from the cross-sectional study: (1) transforming SUA into quintiles and categorizing HUA status (with/without HUA); (2) using a restricted cubic spline method to examine dose-response relationships; (3) applying quasi-Poisson mixed-effects models; (4) adjusting for additional potential covariates; (5) further adjusting for SUA (continuous) in the gout analysis.

In addition to the aforementioned methods, several sensitivity analyses were performed to assess the robustness of the results for transition pattern A: (1) excluding participants who transitioned to different states on the same date; (2) for those who transitioned on the same date, recalculating the entering date of the prior state using two additional time intervals (0.5 year and 1 year) instead of 0.5 day; (3) excluding events occurring within the first, second, and third years of follow-up; (4) further adjusting for SUA (continuous) and HUA (categorical) in the gout analysis. For transition pattern B, we also performed a sensitivity analysis. Specifically, for individuals diagnosed with two or more CMDs on the same day, instead of excluding these participants, we set a random seed and used the R function sample() to randomly select one CMD to represent the earliest diagnosis for that day.

All analyses were performed using R (version 4.4.1). Multi-state models were constructed using the “mstate” package (https://CRAN.R-project.org/package=mstate). Additionally, the restricted cubic spline model with three knots (P10, P50, and P90) was implemented through the “rms” package (version 6.7-1, https://CRAN.R-project.org/package = rms). Statistical tests were two-tailed, and significance was set at P < 0.05.

## Supplementary information

Wu et al_Supplementary Materials(revised)_30Apr2026.

## Data Availability

The data from the UK Biobank utilized in this research is accessible to authorized researchers worldwide via the UK Biobank research portal https://www.ukbiobank.ac.uk/.

## Code availability

The codes employed in this study are available from the authors upon reasonable request. All analyses were conducted using publicly accessible software and packages.

## References

[1] Skou, S. T. et al. Multimorbidity. *Nat. Rev. Dis. Prim.* **8**, 48 (2022). 35835758 10.1038/s41572-022-00376-4 PMC7613517

[2] Busija, L., Lim, K., Szoeke, C., Sanders, K. M. & McCabe, M. P. Do replicable profiles of multimorbidity exist? Systematic review and synthesis. *Eur. J. Epidemiol.* **34**, 1025–1053 (2019). 31624969 10.1007/s10654-019-00568-5

[3] Han, Y. et al. Lifestyle, cardiometabolic disease, and multimorbidity in a prospective Chinese study. *Eur. Heart J.* **42**, 3374–3384 (2021). 34333624 10.1093/eurheartj/ehab413 PMC8423468

[4] Di Angelantonio, E. et al. Association of cardiometabolic multimorbidity with mortality. *JAMA* **314**, 52–60 (2015). 26151266 10.1001/jama.2015.7008 PMC4664176

[5] Dove, A. et al. Cardiometabolic multimorbidity accelerates cognitive decline and dementia progression. *Alzheimers Dement.* **19**, 821–830 (2023). 35708183 10.1002/alz.12708

[6] Steell, L. et al. Multimorbidity clusters and their associations with health-related quality of life in two UK cohorts. *BMC Med.* **23**, 1 (2025). 39773733 10.1186/s12916-024-03811-3 PMC11708164

[7] Khan, U. I. et al. Burden of cardiometabolic diseases and depression in a low-income, urban community in Pakistan: a cross-sectional survey. *BMC Public Health* **25**, 757 (2025). 39994704 10.1186/s12889-025-21939-6 PMC11852865

[8] Li, X. et al. Serum uric acid levels and multiple health outcomes: umbrella review of evidence from observational studies, randomised controlled trials, and Mendelian randomisation studies. *BMJ* **357**, j2376 (2017). 28592419 10.1136/bmj.j2376 PMC5461476

[9] Li, X. et al. MR-PheWAS: exploring the causal effect of SUA level on multiple disease outcomes by using genetic instruments in UK Biobank. *Ann. Rheum. Dis.* **77**, 1039–1047 (2018). 29437585 10.1136/annrheumdis-2017-212534 PMC6029646

[10] Wen, S., Arakawa, H. & Tamai, I. Uric acid in health and disease: from physiological functions to pathogenic mechanisms. *Pharm. Therapeut* **256**, 108615 (2024). 10.1016/j.pharmthera.2024.108615 38382882

[11] Im, P. K. et al. Hyperuricemia, gout and the associated comorbidities in China: findings from a prospective study of 0.5 million adults. *Lancet Reg. Health West Pac.* **58**, 101572 (2025). 40519626 10.1016/j.lanwpc.2025.101572 PMC12167516

[12] Borghi, C., Fogacci, F. & Cicero, A. F. Crystal clear - Part II: the role of uric acid in cardiorenal disease. *Eur. J. Intern. Med.* **142**, 106554 (2025). 40716974 10.1016/j.ejim.2025.07.028

[13] Song, S. et al. Plasma aldosterone concentrations elevation in hypertensive patients: the dual impact on hyperuricemia and gout. *Front. Endocrinol.* **15**, 1424207 (2024). 10.3389/fendo.2024.1424207 PMC11319118 39140032

[14] Quek, A. M. L. et al. Elevated uric acid and impaired triglyceride metabolism predict mortality in women after ischemic stroke. *Free Radic. Biol. Med.* **238**, 542–549 (2025). 40645459 10.1016/j.freeradbiomed.2025.07.015

[15] Li, D., Wang, D., Dai, X., Ni, Y. & Xu, X. Change of serum uric acid and progression of cardiometabolic multimorbidity among middle aged and older adults: a prospective cohort study. *Front. Public Health* **10**, 1012223 (2022). 36388339 10.3389/fpubh.2022.1012223 PMC9644181

[16] Rho, Y. H. et al. Independent impact of gout on the risk of diabetes mellitus among women and men: a population-based, BMI-matched cohort study. *Ann. Rheum. Dis.* **75**, 91–95 (2016). 25277955 10.1136/annrheumdis-2014-205827 PMC4388809

[17] De Vera, M. A., Rahman, M. M., Bhole, V., Kopec, J. A. & Choi, H. K. Independent impact of gout on the risk of acute myocardial infarction among elderly women: a population-based study. *Ann. Rheum. Dis.* **69**, 1162–1164 (2010). 20124358 10.1136/ard.2009.122770 PMC3142935

[18] Chien, K. L. et al. Hyperuricemia as a risk factor on cardiovascular events in Taiwan: the Chin-Shan Community Cardiovascular Cohort Study. *Atherosclerosis* **183**, 147–155 (2005). 16154134 10.1016/j.atherosclerosis.2005.01.018

[19] Yao, Q. & Chen, G. Association of biochemical indicators with multimorbidity in 19,624 older adult individuals with chronic diseases: a study from Jindong District, Jinhua City, China. *Front. Public Health* **13**, 1472415 (2025). 39925752 10.3389/fpubh.2025.1472415 PMC11804261

[20] Sandoval-Plata, G., Nakafero, G., Chakravorty, M., Morgan, K. & Abhishek, A. Association between serum urate, gout and comorbidities: a case-control study using data from the UK Biobank. *Rheumatology* **60**, 3243–3251 (2021). 33313843 10.1093/rheumatology/keaa773

[21] Alonso, A., Rodríguez, L. A., Logroscino, G. & Hernán, M. A. Gout and risk of Parkinson disease: a prospective study. *Neurology* **69**, 1696–1700 (2007). 17954784 10.1212/01.wnl.0000279518.10072.df

[22] Yang, K., Li, J. & Tao, L. Purine metabolism in the development of osteoporosis. *Biomed. Pharmacother.* **155**, 113784 (2022). 36271563 10.1016/j.biopha.2022.113784

[23] Song, S. et al. Serum uric acid and bone health in middle-aged and elderly hypertensive patients: a potential U-shaped association and implications for future fracture risk. *Metabolites* **15**, 15 (2025). 39852358 10.3390/metabo15010015 PMC11766991

[24] Kwon, M. J. et al. Potential association of osteoporosis and not osteoporotic fractures in patients with gout: a longitudinal follow-up study. *Nutrients* **15**, 134 (2022). 36615792 10.3390/nu15010134 PMC9823608

[25] Kodama, S. et al. Association between serum uric acid and development of Type 2 diabetes. *Diab. Care* **32**, 1737–1742 (2009). 10.2337/dc09-0288 PMC2732137 19549729

[26] Bos, M. J., Koudstaal, P. J., Hofman, A., Witteman, J. C. M. & Breteler, M. M. B. Uric acid is a risk factor for myocardial infarction and stroke - The Rotterdam Study. *Stroke* **37**, 1503–1507 (2006). 16675740 10.1161/01.STR.0000221716.55088.d4

[27] Choi, H. K. & Curhan, G. Independent impact of gout on mortality and risk for coronary heart disease. *Circulation* **116**, 894–900 (2007). 17698728 10.1161/CIRCULATIONAHA.107.703389

[28] Zhong, J. et al. Serum uric acid and prognosis of ischemic stroke: cohort study, meta-analysis and Mendelian randomization study. *Eur. Stroke J.* **9**, 235–243 (2024). 37905729 10.1177/23969873231209620 PMC10916819

[29] Zhang, M. et al. Association between uric acid and the prognosis of acute ischemic stroke: a systematic review and meta-analysis. *Nutr. Metab. Cardiovasc Dis.* **31**, 3016–3023 (2021). 34625360 10.1016/j.numecd.2021.07.031

[30] Lee, D. Y. et al. Risk acceleration by gout on major adverse cardiovascular events and all-cause death in patients with diabetes and chronic kidney disease. *Diab. Obes. Metab.* **27**, 1554–1563 (2025). 10.1111/dom.16165 39748223

[31] Li, B. et al. Association of serum uric acid with all-cause and cardiovascular mortality in diabetes. *Diab. Care* **46**, 425–433 (2023). 10.2337/dc22-1339 36490263

[32] He, Y. et al. Serum uric acid levels and risk of cardiovascular disease in type 2 diabetes: results from a cross-sectional study and Mendelian randomization analysis. *Front Endocrinol.* **14**, 1251451 (2023). 10.3389/fendo.2023.1251451 PMC10664243 38027101

[33] San Gabriel, D. E. D. & Slark, J. The association of gout with an increased risk of hypertension and diabetes mellitus among stroke survivors in New Zealand: a cross-sectional study using routinely collected electronic health data. *JRSM Cardiovasc. Dis.* **8**, 2048004019863239 (2019). 31367348 10.1177/2048004019863239 PMC6643175

[34] Simats, A. & Liesz, A. Systemic inflammation after stroke: implications for post-stroke comorbidities. *EMBO Mol. Med.* **14**, e16269 (2022). 35971650 10.15252/emmm.202216269 PMC9449596

[35] Lee, K. W. & Shin, D. Concurrent presence of high serum uric acid and inflammation is associated with increased incidence of type 2 diabetes mellitus in Korean adult population. *Sci. Rep.* **12**, 11000 (2022). 35768559 10.1038/s41598-022-15176-9 PMC9243007

[36] Zhu, Y., Pandya, B. J. & Choi, H. K. Comorbidities of gout and hyperuricemia in the US general population: NHANES 2007-2008. *Am. J. Med.* **125**, 679–87.e1 (2012). 22626509 10.1016/j.amjmed.2011.09.033

[37] Dawson, J. et al. Serum uric acid level, longitudinal blood pressure, renal function, and long-term mortality in treated hypertensive patients. *Hypertension* **62**, 105–111 (2013). 23690348 10.1161/HYPERTENSIONAHA.113.00859

[38] Kuo, C.-F. et al. Elevated risk of mortality among gout patients: a comparison with the National Population in Taiwan. *Jt. Bone Spine* **78**, 577–580 (2011). 10.1016/j.jbspin.2011.01.007 21388850

[39] Lin, Y. et al. The association of C-reactive protein-triglyceride-glucose index with cardiometabolic multimorbidity in middle-aged and older adults: evidence from two cohort studies. *Cardiovasc. Diabetol.* **25**, 90 (2026). 41688996 10.1186/s12933-026-03109-z PMC13005544

[40] Tian, Z. et al. Associations of different insulin resistance-related indices with the incidence and progression trajectory of cardiometabolic multimorbidity: a prospective cohort study from UK biobank. *Cardiovasc. Diabetol.* **24**, 257 (2025). 40533754 10.1186/s12933-025-02819-0 PMC12175334

[41] Dunn, S. E., Perry, W. A. & Klein, S. L. Mechanisms and consequences of sex differences in immune responses. *Nat. Rev. Nephrol.* **20**, 37–55 (2024). 37993681 10.1038/s41581-023-00787-w

[42] Cross, M. et al. Global, regional, and national burden of gout, 1990-2020, and projections to 2050: a systematic analysis of the Global Burden of Disease Study 2021. *Lancet Rheumatol.* **6**, E507–E517 (2024). 38996590 10.1016/S2665-9913(24)00117-6 PMC11263476

[43] Luo, H. et al. Long-term exposure to ambient air pollution is a risk factor for trajectory of cardiometabolic multimorbidity: a prospective study in the UK Biobank. *EBioMedicine* **84**, 104282 (2022). 36174399 10.1016/j.ebiom.2022.104282 PMC9520206

[44] Caraballo, C. et al. Temporal trends in racial and ethnic disparities in multimorbidity prevalence in the United States, 1999-2018. *Am. J. Med.* **135**, 1083–92.e14 (2022). 35472394 10.1016/j.amjmed.2022.04.010

[45] Sudlow, C. et al. UK Biobank: an open access resource for identifying the causes of a wide range of complex diseases of middle and old age. *PLoS Med.* **12**, e1001779 (2015). 25826379 10.1371/journal.pmed.1001779 PMC4380465

[46] Kang, Z. et al. Impaired pulmonary function increases the risk of gout: evidence from a large cohort study in the UK Biobank. *BMC Med.* **22**, 606 (2024). 39736738 10.1186/s12916-024-03836-8 PMC11686989

[47] Chudasama, Y. V. et al. Physical activity, multimorbidity, and life expectancy: a UK Biobank longitudinal study. *BMC Med.* **17**, 108 (2019). 31186007 10.1186/s12916-019-1339-0 PMC6560907

[48] Chen, S. et al. Sociodemographic characteristics and longitudinal progression of multimorbidity: a multistate modelling analysis of a large primary care records dataset in England. *PLoS Med.* **20**, e1004310 (2023). 37922316 10.1371/journal.pmed.1004310 PMC10655992

[49] Zhang, Y. et al. Intake of sugary beverages with chronic conditions and multimorbidity: a prospective cohort study of UK Biobank. *Int. J. Epidemiol.* **52**, 1473–1485 (2023). 37178182 10.1093/ije/dyad057

[50] Putter, H., Fiocco, M. & Geskus, R. B. Tutorial in biostatistics: competing risks and multi-state models. *Stat. Med.* **26**, 2389–2430 (2007). 17031868 10.1002/sim.2712

[51] de Wreede, L. C., Fiocco, M. & Putter, H. The mstate package for estimation and prediction in non- and semi-parametric multi-state and competing risks models. *Comput. Methods Prog. Biomed.* **99**, 261–274 (2010). 10.1016/j.cmpb.2010.01.001 20227129

[52] Wang, Y. X. et al. Association of spontaneous abortion with all cause and cause specific premature mortality: prospective cohort study. *BMJ.* **372**, n530 (2021). 33762255 10.1136/bmj.n530 PMC7988453

