# Supplementary material for: Serum uric acid, gout, cardiometabolic diseases, and multimorbidity: a prospective cohort study using multi-state model
Source: NPJ Aging. 2026 May 25;12(1):112. doi: 10.1038/s41514-026-00413-6 (PMC13482643; doi:10.1038/s41514-026-00413-6)
Supplement: Supplementary file 1 [file 41514_2026_413_MOESM1_ESM.pdf]

Supplementary Materials for

Serum uric acid, gout, cardiometabolic diseases, and multimorbidity: a prospective cohort study using multi-state model

Contents

Figure S1. Associations between serum uric acid, multimorbidity, and 36 chronic conditions in the overall population (N = 468,929). 2
Figure S2. Associations between serum uric acid, multimorbidity, and 36 chronic conditions in females (N = 254,244) and males (N = 214,685). 3
Figure S3. Associations between gout, multimorbidity, and 36 chronic conditions in the overall population (N = 468,929). 4
Figure S4. Associations between gout, multimorbidity, and 36 chronic conditions in females (N = 254,244) and males (N = 214,685). 5
Figure S5. Restricted cubic spline regression analysis of serum uric acid levels and multimorbidity in the overall population, females, and males. 6
Figure S6. Sensitivity analysis of the associations between serum uric acid, gout, and the trajectory of cardiometabolic multimorbidity (transition pattern B). 7
Figure S7. The flowchart of the study population. 8
Table S1. Baseline characteristics by multimorbidity status in females (N = 254,244) and males (N = 214,685) at recruitment. 9
Table S2. Associations between serum uric acid, gout, and the number of chronic conditions assessed using quasi-Poisson mixed-effects models. 11
Table S3. Baseline characteristics by incident disease status in females during follow-up (N = 236,035). 12
Table S4. Baseline characteristics by incident disease status in males during follow-up (N = 182,616). 14
Table S5. Associations between serum uric acid, gout, FCMD, CMM, and mortality assessed using traditional Cox proportional hazards regression models. 16
Table S6. Subgroup analyses of the associations between serum uric acid, gout, and multimorbidity in the cross-sectional study (N = 468,929). 17
Table S7. Sensitivity analyses of associations between serum uric acid and multimorbidity in the cross-sectional study. 19
Table S8. Sensitivity analyses of associations between gout, multimorbidity, and 36 chronic conditions in the cross-sectional study. 20
Table S9. Sensitivity analyses of associations between serum uric acid and the trajectory of cardiometabolic multimorbidity (transition pattern A). 22
Table S10. Sensitivity analyses of associations between gout and the trajectory of cardiometabolic multimorbidity (transition pattern A). 25
Table S11. Details of the variables included in the study. 28
Table S12. 36 chronic conditions included in the cross-sectional study. 35
Supplementary Document 37

Figure S1. Associations between serum uric acid, multimorbidity, and 36 chronic conditions in the overall population (N = 468,929).

Groups	Cases	Total	OR (95% CI)	Pvalue
Multimorbidity	106096	468929	1.267 (1.256, 1.277)	< 0.001
Anaemia	852	468929	1.028 (0.949, 1.113)	0.503
Angina	14954	468929	1.247 (1.225, 1.269)	< 0.001
Anxiety/panic attacks	6287	468929	0.92 (0.892, 0.948)	< 0.001
Asthma	54363	468929	1.096 (1.085, 1.108)	< 0.001
Atrial fibrillation	3426	468929	1.373 (1.325, 1.422)	< 0.001
Bronchiectasis	1059	468929	0.989 (0.922, 1.061)	0.755
Cancer	38614	468929	1.037 (1.025, 1.05)	< 0.001
Chronic kidney disease	811	468929	2.251 (2.121, 2.388)	< 0.001
Chronic obstructive pulmonary disease	1559	468929	1.137 (1.077, 1.2)	< 0.001
Chronic sinusitis	2919	468929	0.991 (0.949, 1.034)	0.667
Cirrhosis	335	468929	1.197 (1.065, 1.346)	0.003
Dementia	114	468929	0.746 (0.599, 0.931)	0.009
Depression	26426	468929	0.995 (0.98, 1.01)	0.512
Diabetes	23665	468929	1.113 (1.097, 1.129)	< 0.001
Eczema or dermatitis	11865	468929	1.031 (1.009, 1.054)	0.005
Epilepsy	3770	468929	0.621 (0.596, 0.647)	< 0.001
Glaucoma	4978	468929	1.007 (0.975, 1.039)	0.686
Heart failure	287	468929	2.28 (2.073, 2.507)	< 0.001
Hepatitis	1997	468929	0.986 (0.937, 1.037)	0.583
Hypertension	124473	468929	1.663 (1.65, 1.676)	< 0.001
Inflammatory bowel disease	3979	468929	0.968 (0.933, 1.005)	0.089
Irritable bowel syndrome	10777	468929	0.971 (0.949, 0.994)	0.013
Meningitis	1878	468929	1.018 (0.966, 1.073)	0.502
Migraine	13602	468929	0.885 (0.867, 0.905)	< 0.001
Multiple sclerosis	1631	468929	0.728 (0.684, 0.776)	< 0.001
Myocardial infarction	10683	468929	1.296 (1.269, 1.323)	< 0.001
Osteoporosis	7471	468929	0.734 (0.713, 0.756)	< 0.001
Parkinson's disease	800	468929	0.555 (0.509, 0.606)	< 0.001
Peripheral vascular disease	866	468929	0.936 (0.864, 1.013)	0.101
Prostate problem	791	468929	0.911 (0.842, 0.986)	0.02
Rheumatoid arthritis	5287	468929	1.002 (0.97, 1.034)	0.923
Schizophrenia	556	468929	0.943 (0.859, 1.037)	0.227
Stroke	6198	468929	1.234 (1.201, 1.268)	< 0.001
Thyroid problem	26630	468929	1.164 (1.148, 1.182)	< 0.001
Tuberculosis	2400	468929	0.997 (0.952, 1.044)	0.893
Vestibular disorder	4009	468929	1.016 (0.981, 1.054)	0.375

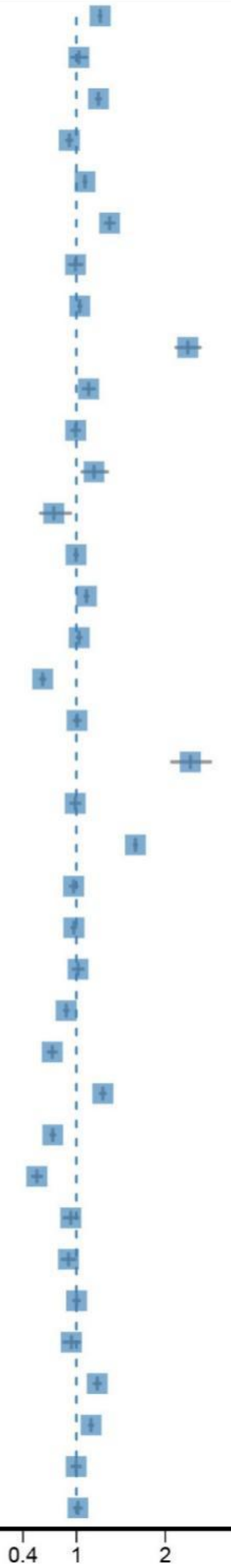

Note: The model was adjusted for sex (male/female), age at recruitment (continuous), ethnic background (white British/non-white British), and the Townsend deprivation index (1st/2nd/3rd/4th/5th quintile). Associations are reported per standard deviation increase in SUA levels. CI, confidence interval; OR, odds ratio.

Figure S2. Associations between serum uric acid, multimorbidity, and 36 chronic conditions in females (N = 254,244) and males (N = 214,685).

Chronic conditions	Cases/Total_female	OR (95% CI)	Cases/Total_male	OR (95% CI)	P for interaction
Multimorbidity	59089/254244	1.406 (1.39, 1.422)	47007/214685	1.151 (1.138, 1.165)	< 0.001
Anaemia	629/254244	1.022 (0.927, 1.127)	223/214685	1.131 (0.982, 1.302)	0.067
Angina	4864/254244	1.421 (1.378, 1.465)	10090/214685	1.166 (1.141, 1.191)	< 0.001
Anxiety/panic attacks	4046/254244	0.92 (0.884, 0.957)	2241/214685	0.912 (0.87, 0.956)	0.612
Asthma	31344/254244	1.143 (1.126, 1.159)	23019/214685	1.04 (1.025, 1.056)	< 0.001
Atrial fibrillation	1076/254244	1.488 (1.398, 1.583)	2350/214685	1.313 (1.257, 1.37)	< 0.001
Bronchiectasis	693/254244	0.927 (0.846, 1.015)	366/214685	1.083 (0.969, 1.212)	0.043
Cancer	24747/254244	1.083 (1.066, 1.1)	13867/214685	0.993 (0.974, 1.013)	< 0.001
Chronic kidney disease	355/254244	2.601 (2.387, 2.834)	456/214685	2.021 (1.863, 2.192)	< 0.001
Chronic obstructive pulmonary disease	797/254244	1.156 (1.069, 1.25)	762/214685	1.126 (1.044, 1.214)	0.72
Chronic sinusitis	1759/254244	1.016 (0.959, 1.076)	1160/214685	0.962 (0.901, 1.027)	0.244
Cirrhosis	171/254244	1.388 (1.18, 1.633)	164/214685	1.02 (0.863, 1.205)	0.006
Dementia	51/254244	0.703 (0.492, 1.005)	63/214685	0.79 (0.596, 1.047)	0.512
Depression	17367/254244	1.044 (1.025, 1.064)	9059/214685	0.931 (0.909, 0.953)	< 0.001
Diabetes	8810/254244	1.693 (1.655, 1.732)	14855/214685	0.858 (0.842, 0.875)	0.512
Eczema or dermatitis	6502/254244	1.064 (1.032, 1.097)	5363/214685	1.01 (0.98, 1.041)	0.07
Epilepsy	1903/254244	0.702 (0.66, 0.746)	1867/214685	0.561 (0.531, 0.593)	< 0.001
Glaucoma	2355/254244	1.044 (0.995, 1.095)	2623/214685	0.973 (0.932, 1.016)	0.018
Heart failure	102/254244	2.664 (2.298, 3.09)	185/214685	2.083 (1.84, 2.359)	0.017
Hepatitis	977/254244	0.953 (0.882, 1.031)	1020/214685	0.999 (0.933, 1.07)	0.605
Hypertension	59370/254244	1.947 (1.924, 1.971)	65103/214685	1.455 (1.44, 1.471)	< 0.001
Inflammatory bowel disease	2198/254244	1.006 (0.955, 1.06)	1781/214685	0.927 (0.879, 0.977)	0.022
Irritable bowel syndrome	8033/254244	0.984 (0.957, 1.012)	2744/214685	0.93 (0.891, 0.97)	0.013
Meningitis	957/254244	1.055 (0.976, 1.14)	921/214685	0.986 (0.917, 1.06)	0.2
Migraine	10623/254244	0.886 (0.864, 0.908)	2979/214685	0.885 (0.849, 0.922)	0.985
Multiple sclerosis	1189/254244	0.776 (0.719, 0.837)	442/214685	0.626 (0.559, 0.7)	0.001
Myocardial infarction	2093/254244	1.497 (1.432, 1.566)	8590/214685	1.245 (1.216, 1.274)	< 0.001
Osteoporosis	6598/254244	0.716 (0.693, 0.739)	873/214685	0.816 (0.757, 0.881)	0.006
Parkinson's disease	289/254244	0.557 (0.475, 0.652)	511/214685	0.552 (0.497, 0.613)	0.842
Peripheral vascular disease	527/254244	0.951 (0.854, 1.059)	339/214685	0.959 (0.851, 1.079)	0.495
Prostate problem	0/254244	NA	791/214685	0.911 (0.842, 0.986)	< 0.001
Rheumatoid arthritis	3674/254244	1.017 (0.978, 1.058)	1613/214685	0.978 (0.926, 1.033)	0.313
Schizophrenia	176/254244	1.297 (1.102, 1.528)	380/214685	0.811 (0.724, 0.909)	< 0.001
Stroke	2489/254244	1.357 (1.3, 1.416)	3709/214685	1.172 (1.132, 1.213)	< 0.001
Thyroid problem	22517/254244	1.188 (1.169, 1.208)	4113/214685	1.076 (1.04, 1.113)	< 0.001
Tuberculosis	1305/254244	0.999 (0.936, 1.066)	1095/214685	0.987 (0.924, 1.054)	0.64
Vestibular disorder	1994/254244	1.025 (0.971, 1.081)	2015/214685	1.011 (0.963, 1.062)	0.763

Female

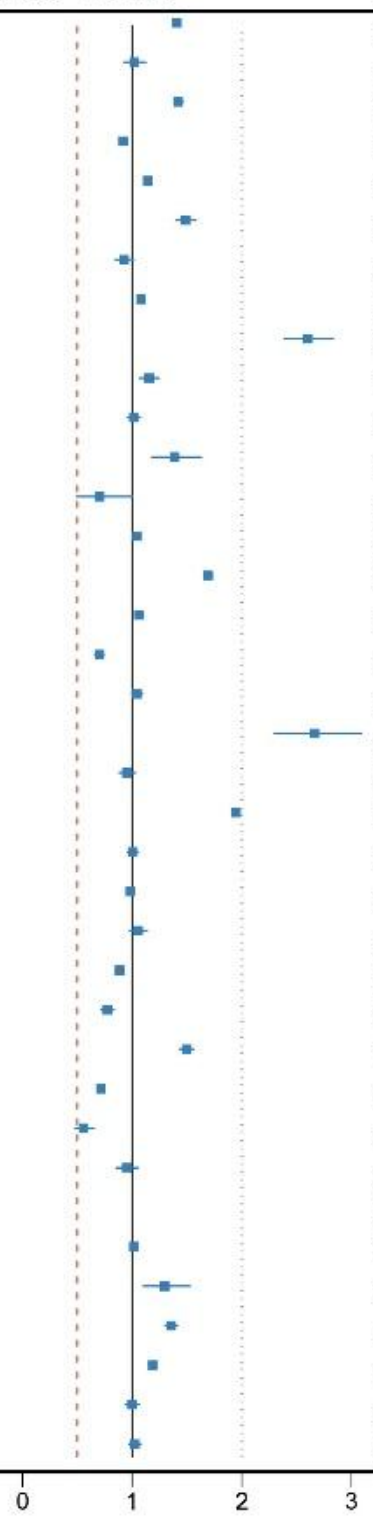

Male

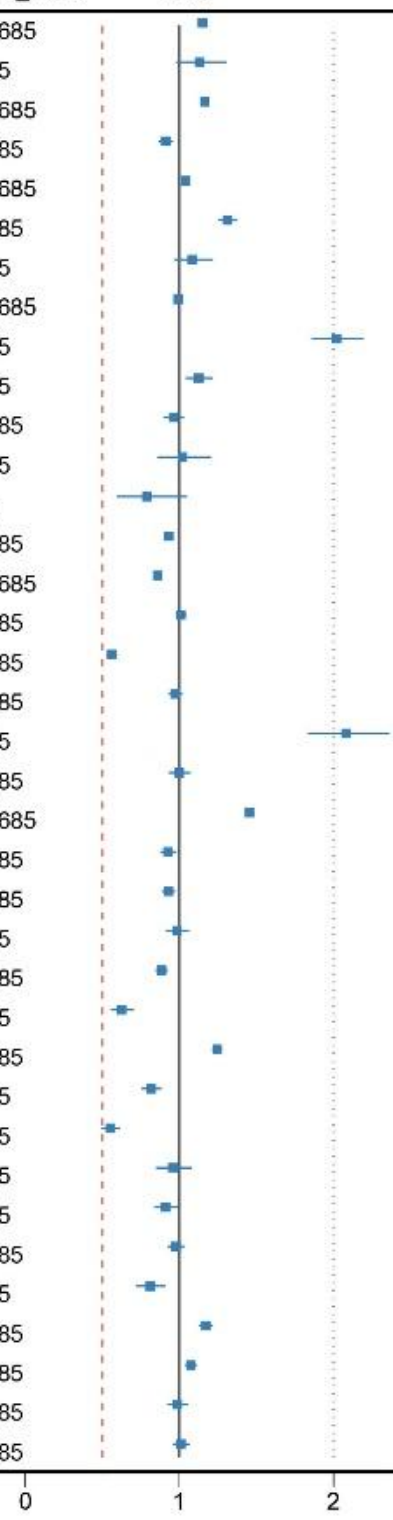

Note: The model was adjusted for age at recruitment (continuous), ethnic background (white British/non-white British), and the Townsend deprivation index (1st/2nd/3rd/4th/5th quintile). Associations are reported per standard deviation increase in SUA levels. CI, confidence interval; OR, odds ratio.

Figure S3. Associations between gout, multimorbidity, and 36 chronic conditions in the overall population (N = 468,929).

Groups	Cases	Total	OR (95% CI)	Pvalue
Multimorbidity	3807	9945	1.946 (1.865, 2.031)	< 0.001
Anaemia	28	9945	2.593 (1.76, 3.821)	< 0.001
Angina	853	9945	1.633 (1.516, 1.76)	< 0.001
Anxiety/panic attacks	101	9945	0.943 (0.772, 1.151)	0.564
Asthma	1179	9945	1.145 (1.076, 1.218)	< 0.001
Atrial fibrillation	238	9945	1.992 (1.739, 2.281)	< 0.001
Bronchiectasis	30	9945	1.361 (0.94, 1.969)	0.103
Cancer	845	9945	1.08 (1.004, 1.161)	0.038
Chronic kidney disease	129	9945	7.481 (6.123, 9.142)	< 0.001
Chronic obstructive pulmonary disease	43	9945	0.942 (0.693, 1.281)	0.703
Chronic sinusitis	51	9945	0.898 (0.679, 1.189)	0.453
Cirrhosis	11	9945	1.366 (0.742, 2.513)	0.316
Dementia	0	9945	0 (0, 0)	0.973
Depression	487	9945	1.155 (1.052, 1.268)	0.003
Diabetes	1340	9945	1.984 (1.867, 2.109)	< 0.001
Eczema or dermatitis	243	9945	1.046 (0.919, 1.192)	0.495
Epilepsy	52	9945	0.605 (0.459, 0.798)	< 0.001
Glaucoma	152	9945	1.023 (0.867, 1.206)	0.788
Heart failure	44	9945	5.388 (3.855, 7.531)	< 0.001
Hepatitis	32	9945	0.662 (0.465, 0.941)	0.022
Hypertension	5514	9945	2.651 (2.543, 2.764)	< 0.001
Inflammatory bowel disease	78	9945	0.925 (0.737, 1.161)	0.501
Irritable bowel syndrome	136	9945	0.934 (0.786, 1.11)	0.438
Meningitis	42	9945	0.988 (0.725, 1.346)	0.937
Migraine	115	9945	0.73 (0.606, 0.88)	0.001
Multiple sclerosis	17	9945	0.775 (0.478, 1.255)	0.3
Myocardial infarction	685	9945	1.593 (1.467, 1.729)	< 0.001
Osteoporosis	63	9945	0.732 (0.569, 0.942)	0.015
Parkinson's disease	15	9945	0.514 (0.308, 0.86)	0.011
Peripheral vascular disease	21	9945	1.244 (0.801, 1.93)	0.331
Prostate problem	37	9945	0.927 (0.665, 1.291)	0.653
Rheumatoid arthritis	127	9945	1.365 (1.139, 1.634)	0.001
Schizophrenia	5	9945	0.321 (0.133, 0.776)	0.012
Stroke	321	9945	1.663 (1.481, 1.869)	< 0.001
Thyroid problem	386	9945	1.343 (1.208, 1.493)	< 0.001
Tuberculosis	70	9945	1.12 (0.88, 1.426)	0.358
Vestibular disorder	103	9945	1.032 (0.846, 1.259)	0.757

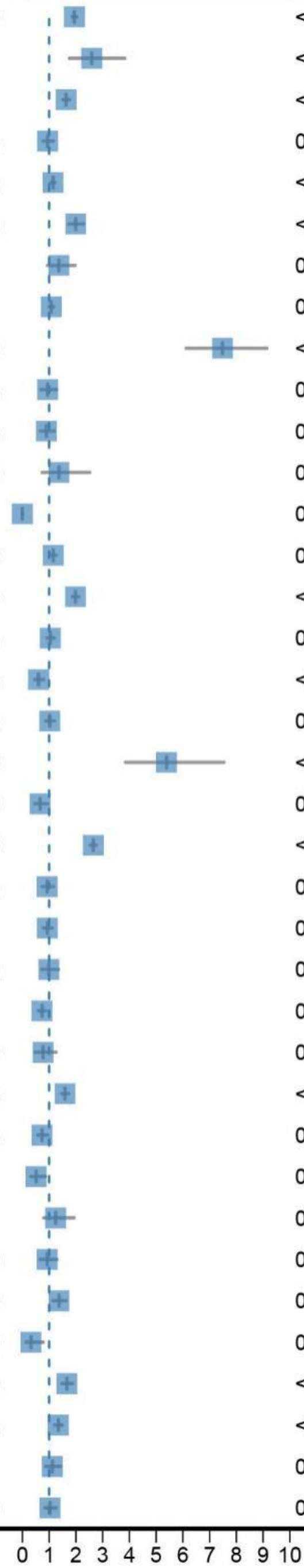

Note: The model was adjusted for sex (male/female), age at recruitment (continuous), ethnic background (white British/non-white British), and the Townsend deprivation index (1st/2nd/3rd/4th/5th quintile). CI, confidence interval; OR, odds ratio.

Figure S4. Associations between gout, multimorbidity, and 36 chronic conditions in females (N = 254,244) and males (N = 214,685).

Chronic conditions	Cases/Total_female	Female	OR (95% CI)	Cases/Total_male	Male	OR (95% CI)	P for interaction
Multimorbidity	523/1022		2.758 (2.433, 3.125)	3284/8923		1.843 (1.761, 1.929)	< 0.001
Anaemia	6/1022		2.716 (1.21, 6.098)	22/8923		2.209 (1.418, 3.439)	0.857
Angina	79/1022		2.7 (2.132, 3.42)	774/8923		1.569 (1.451, 1.696)	< 0.001
Anxiety/panic attacks	16/1022		1.002 (0.611, 1.645)	85/8923		0.946 (0.76, 1.177)	0.729
Asthma	184/1022		1.602 (1.365, 1.881)	995/8923		1.098 (1.026, 1.175)	< 0.001
Atrial fibrillation	17/1022		2.635 (1.622, 4.282)	221/8923		1.976 (1.717, 2.276)	0.273
Bronchiectasis	7/1022		1.81 (0.856, 3.827)	23/8923		1.277 (0.836, 1.951)	0.401
Cancer	148/1022		1.265 (1.061, 1.508)	697/8923		1.018 (0.939, 1.102)	0.155
Chronic kidney disease	24/1022		15.833 (10.365, 24.187)	105/8923		6.533 (5.232, 8.157)	0.012
Chronic obstructive pulmonary disease	8/1022		1.606 (0.796, 3.24)	35/8923		0.852 (0.606, 1.198)	0.165
Chronic sinusitis	7/1022		0.961 (0.456, 2.025)	44/8923		0.883 (0.653, 1.196)	0.857
Cirrhosis	2/1022		2.349 (0.58, 9.508)	9/8923		1.3 (0.662, 2.555)	0.439
Dementia	0/1022		0 (0, 0)	0/8923		0 (0, 0)	1
Depression	80/1022		1.233 (0.98, 1.551)	407/8923		1.131 (1.021, 1.253)	0.626
Diabetes	180/1022		4.575 (3.875, 5.401)	1160/8923		1.796 (1.683, 1.917)	< 0.001
Eczema or dermatitis	33/1022		1.463 (1.033, 2.073)	210/8923		0.985 (0.857, 1.134)	0.081
Epilepsy	6/1022		0.782 (0.35, 1.748)	46/8923		0.588 (0.438, 0.789)	0.449
Glaucoma	28/1022		2.05 (1.402, 2.998)	124/8923		0.92 (0.767, 1.104)	< 0.001
Heart failure	5/1022		8.776 (3.542, 21.746)	39/8923		5.105 (3.573, 7.294)	0.599
Hepatitis	7/1022		1.611 (0.763, 3.401)	25/8923		0.58 (0.389, 0.863)	0.024
Hypertension	591/1022		3.416 (3.005, 3.883)	4923/8923		2.58 (2.469, 2.696)	< 0.001
Inflammatory bowel disease	16/1022		1.781 (1.084, 2.926)	62/8923		0.824 (0.638, 1.063)	0.01
Irritable bowel syndrome	41/1022		1.285 (0.939, 1.758)	95/8923		0.853 (0.694, 1.049)	0.022
Meningitis	7/1022		1.796 (0.851, 3.792)	35/8923		0.905 (0.644, 1.271)	0.127
Migraine	28/1022		0.719 (0.494, 1.048)	87/8923		0.726 (0.585, 0.9)	0.968
Multiple sclerosis	7/1022		1.603 (0.76, 3.383)	10/8923		0.574 (0.306, 1.077)	0.036
Myocardial infarction	35/1022		2.722 (1.931, 3.837)	650/8923		1.559 (1.433, 1.695)	0.004
Osteoporosis	27/1022		0.668 (0.455, 0.982)	36/8923		0.841 (0.601, 1.175)	0.766
Parkinson's disease	0/1022		0 (0, 0)	15/8923		0.549 (0.328, 0.918)	0.204
Peripheral vascular disease	5/1022		2.438 (1.006, 5.906)	16/8923		0.993 (0.6, 1.643)	0.197
Prostate problem	NA		NA	37/8923		0.927 (0.665, 1.291)	1
Rheumatoid arthritis	30/1022		1.655 (1.148, 2.386)	97/8923		1.281 (1.042, 1.576)	0.272
Schizophrenia	1/1022		1.223 (0.171, 8.77)	4/8923		0.28 (0.104, 0.752)	0.221
Stroke	34/1022		2.493 (1.763, 3.524)	287/8923		1.582 (1.399, 1.79)	0.058
Thyroid problem	151/1022		1.506 (1.265, 1.792)	235/8923		1.257 (1.1, 1.437)	0.197
Tuberculosis	10/1022		1.316 (0.703, 2.462)	60/8923		1.104 (0.849, 1.434)	0.554
Vestibular disorder	12/1022		1.318 (0.744, 2.335)	91/8923		0.999 (0.808, 1.234)	0.407
		0 5 10 15 20			0 2 4 6 8		

Note: The model was adjusted for age at recruitment (continuous), ethnic background (white British/non-white British), and the Townsend deprivation index (1st/2nd/3rd/4th/5th quintile). CI, confidence interval; OR, odds ratio.

Figure S5. Restricted cubic spline regression analysis of serum uric acid levels and multimorbidity in the overall population, females, and males.

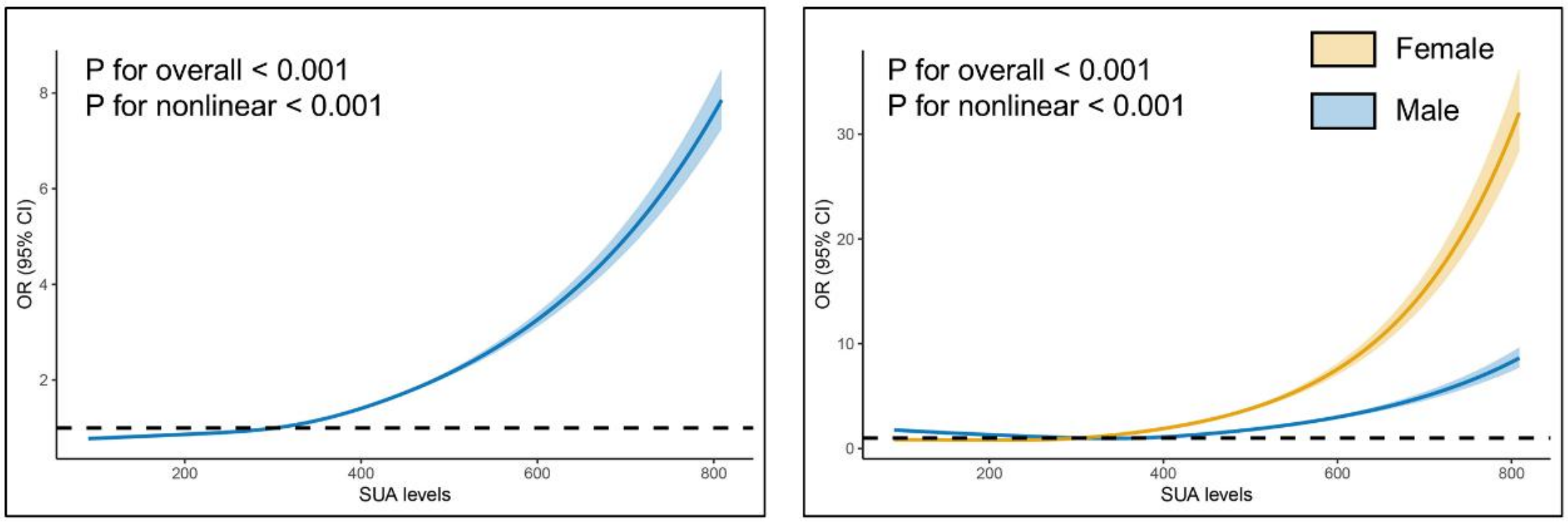

Note: The model was adjusted for sex (male/female), age at recruitment (continuous), ethnic background (white British/non-white British), and the Townsend deprivation index (1st/2nd/3rd/4th/5th quintile). For sex-specific analyses, sex was not included in the model. CI, confidence interval; OR, odds ratio; SUA, serum uric acid.

Figure S6. Sensitivity analysis of the associations between serum uric acid, gout, and the trajectory of cardiometabolic multimorbidity (transition pattern B).

Exposures	Cases	Total	HR (95% CI)	Pvalue
**SUA**				
Baseline -> FCMD				
Baseline -> T2D	15,259	418,651	1.695 (1.668, 1.724)	< 0.001
Baseline -> IHD	26,213	418,651	1.178 (1.162, 1.195)	< 0.001
Baseline -> Stroke	10,570	418,651	1.07 (1.047, 1.095)	< 0.001
FCMD -> CMM				
T2D -> CMM	2,117	15,259	1.021 (0.976, 1.067)	0.368
IHD -> CMM	2,893	26,213	1.187 (1.140, 1.235)	< 0.001
Stroke -> CMM	1,373	10,570	1.206 (1.139, 1.278)	< 0.001
Baseline -> Death	16,810	418,651	1.046 (1.027, 1.065)	< 0.001
FCMD -> Death				
T2D -> Death	1,346	15,259	0.945 (0.894, 1.000)	0.05
IHD -> Death	3,749	26,213	1.028 (0.982, 1.077)	0.24
Stroke -> Death	2,402	10,570	0.958 (0.912, 1.005)	0.079
CMM -> Death	1,572	6,383	1.052 (0.967, 1.145)	0.235
**Gout**				
Baseline -> FCMD				
Baseline -> T2D	655	7,299	2.09 (1.93, 2.26)	< 0.001
Baseline -> IHD	841	7,299	1.24 (1.15, 1.33)	< 0.001
Baseline -> Stroke	287	7,299	1.17 (1.04, 1.32)	0.009
FCMD -> CMM				
T2D -> CMM	108	655	1.03 (0.849, 1.26)	0.737
IHD -> CMM	121	841	1.27 (1.06, 1.53)	0.011
Stroke -> CMM	57	287	1.45 (1.11, 1.9)	0.007
Baseline -> Death	429	7,299	1.12 (1.02, 1.23)	0.021
FCMD -> Death				
T2D -> Death	73	655	0.982 (0.767, 1.26)	0.887
IHD -> Death	141	841	1.02 (0.822, 1.27)	0.859
Stroke -> Death	76	287	1.08 (0.838, 1.39)	0.56
CMM -> Death	73	286	0.742 (0.487, 1.13)	0.163

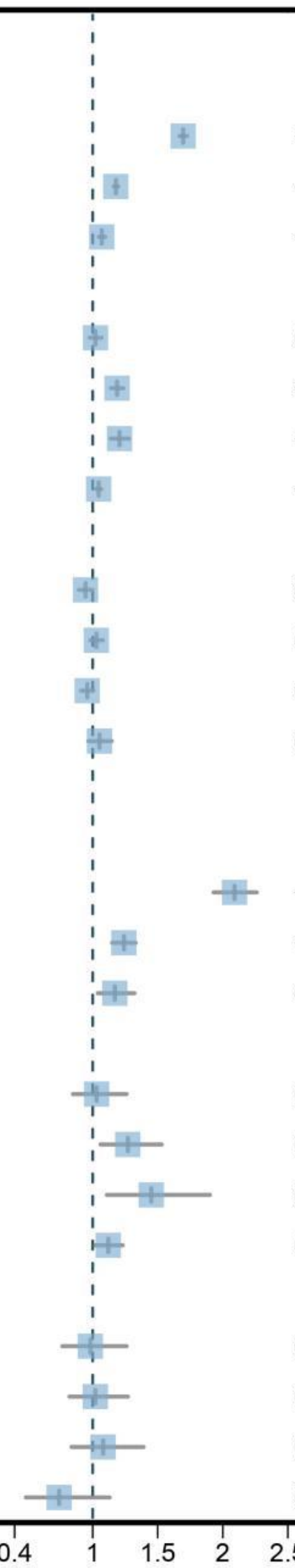

Note: For individuals diagnosed with two or more CMDs on the same day (n = 1,225), instead of excluding these participants, one CMD was randomly selected to represent the earliest diagnosis for that day, and the data were re-analyzed. The model was adjusted for sex (male/female), age at recruitment (continuous), ethnic background (white British/non-white British), and the Townsend deprivation index (1st/2nd/3rd/4th/5th quintile). CI, confidence interval; CMD, cardiometabolic diseases; CMM, cardiometabolic multimorbidity; FCMD, first cardiometabolic disease; HR, hazard ratio; IHD, ischemic heart disease; SUA, serum uric acid; T2D, type 2 diabetes.

Figure S7. The flowchart of the study population.

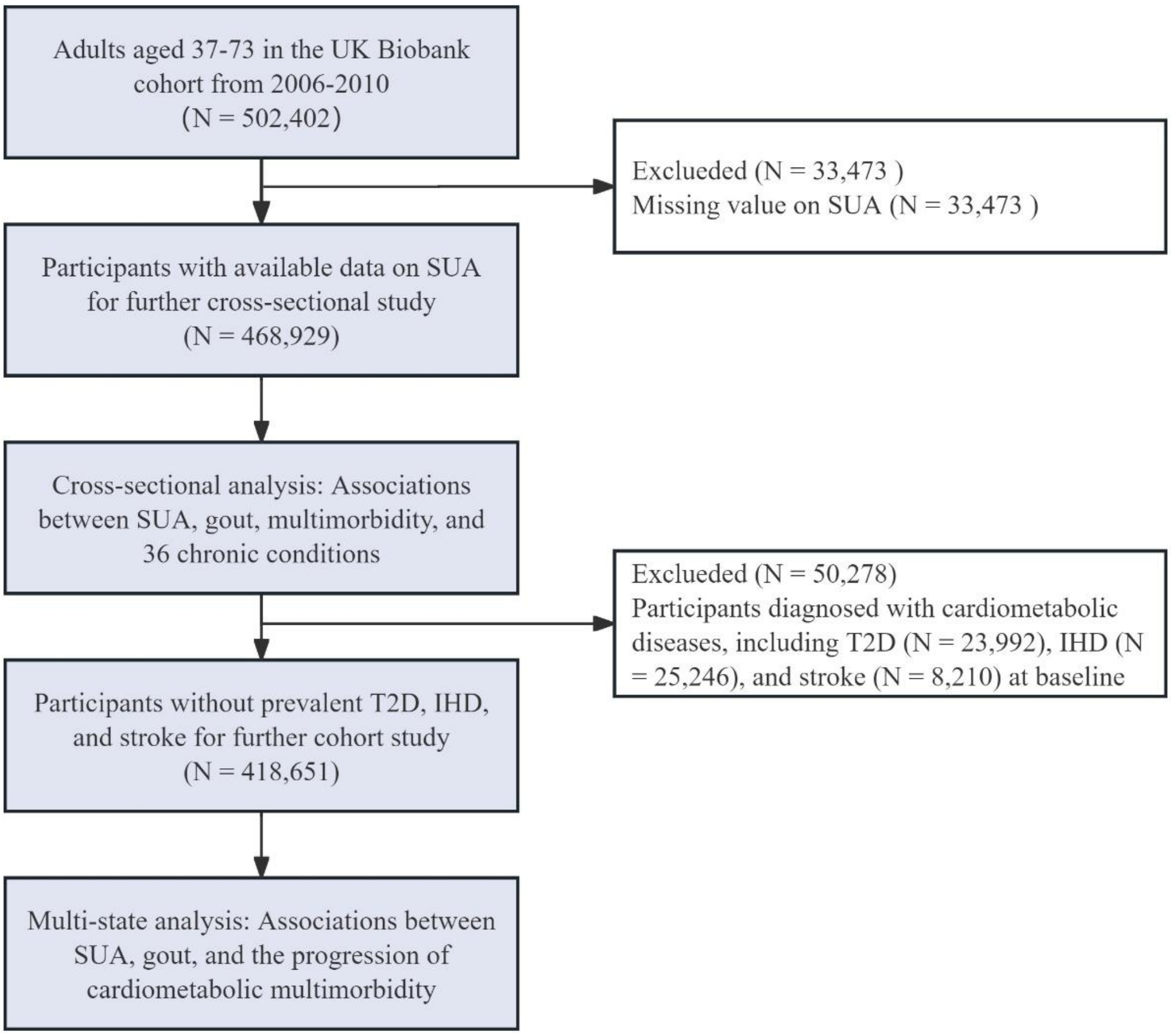

Note: IHD, ischemic heart disease; SUA, serum uric acid; T2D, type 2 diabetes.

Table S1. Baseline characteristics by multimorbidity status in females (N = 254,244) and males (N = 214,685) at recruitment.

	Female		Male	
	**Without multimorbidity**	**With multimorbidity**	**Without multimorbidity**	**With multimorbidity**
Number	195155	59089	167678	47007
Male, N (%)	0 (0.0)	0 (0.0)	167678 (100.0)	47007 (100.0)
Age, mean (SD), years	55.64 (8.02)	58.74 (7.45)	55.89 (8.24)	59.77 (7.27)
Ethnicity, N (%)				
White British	171708 (88.0)	52276 (88.5)	148293 (88.4)	41704 (88.7)
Non-white British	22688 (11.6)	6555 (11.1)	18487 (11.0)	5012 (10.7)
Missing	759 (0.4)	258 (0.4)	898 (0.5)	291 (0.6)
Townsend deprivation index, N (%)				
1st quintile (least deprived)	40245 (20.6)	10507 (17.8)	34808 (20.8)	8308 (17.7)
2nd quintile	39933 (20.5)	11013 (18.6)	34208 (20.4)	8665 (18.4)
3th quintile	39918 (20.5)	11532 (19.5)	33537 (20.0)	8695 (18.5)
4th quintile	39280 (20.1)	12194 (20.6)	32906 (19.6)	9396 (20.0)
5th quintile (most deprived)	35779 (18.3)	13843 (23.4)	32219 (19.2)	11943 (25.4)
Drinking frequency, N (%)				
Frequent	127222 (65.2)	31271 (52.9)	132348 (78.9)	33485 (71.2)
Infrequent	51715 (26.5)	19448 (32.9)	25784 (15.4)	8997 (19.1)
Never	15823 (8.1)	8236 (13.9)	9174 (5.5)	4392 (9.3)
Missing	395 (0.2)	134 (0.2)	372 (0.2)	133 (0.3)
Smoking status, N (%)				
Never	118118 (60.5)	32609 (55.2)	85916 (51.2)	18680 (39.7)

	Female		Male	
	Without multimorbidity	With multimorbidity	Without multimorbidity	With multimorbidity
Previous	59215 (30.3)	20478 (34.7)	60065 (35.8)	22248 (47.3)
Current	16914 (8.7)	5651 (9.6)	20901 (12.5)	5755 (12.2)
Missing	908 (0.5)	351 (0.6)	796 (0.5)	324 (0.7)
Education, N (%)				
College or above	64296 (32.9)	15033 (25.4)	59592 (35.5)	12944 (27.5)
High school	99823 (51.2)	29252 (49.5)	80688 (48.1)	21743 (46.3)
Less than high school	28961 (14.8)	13974 (23.6)	25434 (15.2)	11595 (24.7)
Missing	2075 (1.1)	830 (1.4)	1964 (1.2)	725 (1.5)
Body mass index, N (%)				
<25.0 Kg/m2	84111 (43.1)	16628 (28.1)	45769 (27.3)	8033 (17.1)
25-29.9 Kg/m2	72385 (37.1)	21609 (36.6)	85337 (50.9)	21297 (45.3)
≥30.0 Kg/m2	38659 (19.8)	20852 (35.3)	36572 (21.8)	17677 (37.6)
Urate, mean (SD), µmmol/L	265.43 (62.00)	289.40 (75.78)	352.34 (69.10)	362.04 (80.30)
Gout at baseline, N (%)	499 (0.3)	523 (0.9)	5639 (3.4)	3284 (7.0)

Note: Categorical data presented as numbers (percentages) and continuous data presented as mean (standard deviation). SD, standard deviation.

Table S2. Associations between serum uric acid, gout, and the number of chronic conditions assessed using quasi-Poisson mixed-effects models.

Exposures	RR (95% CI)			P for interaction
	Overall	Female	Male	
SUA	1.142 (1.138, 1.146)	1.2 (1.194, 1.206)	1.092 (1.086, 1.098)	P < 0.001
Gout	1.431 (1.405, 1.457)	2.758 (2.433, 3.125)	1.692 (1.611, 1.775)	P < 0.001

Note: The model was adjusted for sex (male/female), age at recruitment (continuous), ethnic background (white British/non-white British), and the Townsend deprivation index (1st/2nd/3rd/4th/5th quintile). For sex-specific analyses, sex was not included in the model. CI, confidence interval; RR, relative risk; SUA, serum uric acid.

Table S3. Baseline characteristics by incident disease status in females during follow-up (N = 236,035).

	CMD free	FCMD survivor	CMM survivor	Dead without CMD	Dead with FCMD	Dead with CMM
Number	205400	17008	1927	8279	2850	571
Age, mean (SD), years	55.46 (7.97)	59.26 (7.26)	60.71 (6.74)	60.41 (6.81)	62.10 (6.11)	62.95 (5.49)
Ethnicity, N (%)						
White British	181638 (88.4)	14741 (86.7)	1642 (85.2)	7544 (91.1)	2582 (90.6)	502 (87.9)
Non-white British	23020 (11.2)	2177 (12.8)	273 (14.2)	694 (8.4)	258 (9.1)	67 (11.7)
Missing	742 (0.4)	90 (0.5)	12 (0.6)	41 (0.5)	10 (0.4)	2 (0.4)
Townsend deprivation index, N (%)						
1st quintile (least deprived)	42683 (20.8)	3055 (18.0)	263 (13.6)	1612 (19.5)	482 (16.9)	84 (14.7)
2nd quintile	42244 (20.6)	3163 (18.6)	328 (17.0)	1669 (20.2)	461 (16.2)	98 (17.2)
3th quintile	42110 (20.5)	3316 (19.5)	383 (19.9)	1596 (19.3)	576 (20.2)	102 (17.9)
4th quintile	41314 (20.1)	3533 (20.8)	390 (20.2)	1669 (20.2)	612 (21.5)	123 (21.5)
5th quintile (most deprived)	37049 (18.0)	3941 (23.2)	563 (29.2)	1733 (20.9)	719 (25.2)	164 (28.7)
Drinking frequency, N (%)						
Frequent	133898 (65.2)	9097 (53.5)	855 (44.4)	4936 (59.6)	1529 (53.6)	285 (49.9)
Infrequent	54658 (26.6)	5623 (33.1)	733 (38.0)	2379 (28.7)	884 (31.0)	191 (33.5)
Never	16481 (8.0)	2229 (13.1)	333 (17.3)	946 (11.4)	431 (15.1)	94 (16.5)
Missing	363 (0.2)	59 (0.3)	6 (0.3)	18 (0.2)	6 (0.2)	1 (0.2)
Smoking status, N (%)						
Never	125032 (60.9)	9432 (55.5)	1014 (52.6)	4081 (49.3)	1335 (46.8)	252 (44.1)
Previous	63008 (30.7)	5526 (32.5)	630 (32.7)	2885 (34.8)	947 (33.2)	185 (32.4)
Current	16508 (8.0)	1933 (11.4)	271 (14.1)	1246 (15.1)	544 (19.1)	125 (21.9)

	CMD free	FCMD survivor	CMM survivor	Dead without CMD	Dead with FCMD	Dead with CMM
Missing	852 (0.4)	117 (0.7)	12 (0.6)	67 (0.8)	24 (0.8)	9 (1.6)
Education, N (%)						
College or above	68690 (33.4)	3835 (22.5)	334 (17.3)	2182 (26.4)	599 (21.0)	113 (19.8)
High school	105482 (51.4)	8685 (51.1)	925 (48.0)	3933 (47.5)	1274 (44.7)	223 (39.1)
Less than high school	29186 (14.2)	4193 (24.7)	633 (32.8)	2049 (24.7)	924 (32.4)	221 (38.7)
Missing	2042 (1.0)	295 (1.7)	35 (1.8)	115 (1.4)	53 (1.9)	14 (2.5)
BMI, N (%)						
<25.0 Kg/m2	88329 (43.0)	4374 (25.7)	347 (18.0)	3177 (38.4)	943 (33.1)	149 (26.1)
25-29.9 Kg/m2	76213 (37.1)	6398 (37.6)	729 (37.8)	3129 (37.8)	1047 (36.7)	190 (33.3)
≥30.0 Kg/m2	40858 (19.9)	6236 (36.7)	851 (44.2)	1973 (23.8)	860 (30.2)	232 (40.6)
Urate, mean (SD), µmmol/L	265.25 (62.12)	292.32 (72.00)	308.63 (76.89)	280.48 (70.70)	292.21 (76.72)	320.60 (86.30)
Gout at baseline, N (%)	516 (0.3)	110 (0.6)	24 (1.2)	52 (0.6)	29 (1.0)	8 (1.4)

Note: Categorical data presented as numbers (percentages) and continuous data presented as mean (standard deviation). CMD, cardiometabolic disease; CMM, cardiometabolic multimorbidity; FCMD, first cardiometabolic disease; SD, standard deviation.

Table S4. Baseline characteristics by incident disease status in males during follow-up (N = 182,616).

	CMD free	FCMD survivor	CMM survivor	Dead without CMD	Dead with FCMD	Dead with CMM
Number	144399	21154	2884	8531	4647	1001
Age, mean (SD), years	55.04 (8.21)	58.58 (7.42)	59.89 (6.98)	61.07 (6.73)	61.72 (6.42)	62.67 (5.96)
Ethnicity, N (%)						
White British	128039 (88.7)	18685 (88.3)	2458 (85.2)	7868 (92.2)	4213 (90.7)	873 (87.2)
Non-white British	15629 (10.8)	2341 (11.1)	399 (13.8)	622 (7.3)	407 (8.8)	116 (11.6)
Missing	731 (0.5)	128 (0.6)	27 (0.9)	41 (0.5)	27 (0.6)	12 (1.2)
Townsend deprivation index, N (%)						
1st quintile (least deprived)	30595 (21.2)	4147 (19.6)	504 (17.5)	1631 (19.1)	802 (17.3)	162 (16.2)
2nd quintile	29776 (20.6)	4174 (19.7)	512 (17.8)	1646 (19.3)	831 (17.9)	163 (16.3)
3th quintile	28892 (20.0)	4187 (19.8)	542 (18.8)	1617 (19.0)	858 (18.5)	185 (18.5)
4th quintile	28399 (19.7)	4148 (19.6)	543 (18.8)	1638 (19.2)	920 (19.8)	211 (21.1)
5th quintile (most deprived)	26737 (18.5)	4498 (21.3)	783 (27.1)	1999 (23.4)	1236 (26.6)	280 (28.0)
Drinking frequency, N (%)						
Frequent	114993 (79.6)	16026 (75.8)	2022 (70.1)	6625 (77.7)	3509 (75.5)	716 (71.5)
Infrequent	21696 (15.0)	3652 (17.3)	566 (19.6)	1312 (15.4)	754 (16.2)	171 (17.1)
Never	7439 (5.2)	1416 (6.7)	288 (10.0)	575 (6.7)	362 (7.8)	106 (10.6)
Missing	271 (0.2)	60 (0.3)	8 (0.3)	19 (0.2)	22 (0.5)	8 (0.8)
Smoking status, N (%)						
Never	77272 (53.5)	9453 (44.7)	1147 (39.8)	3164 (37.1)	1597 (34.4)	286 (28.6)
Previous	50420 (34.9)	8657 (40.9)	1226 (42.5)	3594 (42.1)	1960 (42.2)	476 (47.6)
Current	16122 (11.2)	2926 (13.8)	489 (17.0)	1720 (20.2)	1046 (22.5)	227 (22.7)

	CMD free	FCMD survivor	CMM survivor	Dead without CMD	Dead with FCMD	Dead with CMM
Missing	585 (0.4)	118 (0.6)	22 (0.8)	53 (0.6)	44 (0.9)	12 (1.2)
Education, N (%)						
College or above	54422 (37.7)	6134 (29.0)	704 (24.4)	2313 (27.1)	1161 (25.0)	194 (19.4)
High school	69691 (48.3)	10299 (48.7)	1306 (45.3)	3925 (46.0)	2043 (44.0)	419 (41.9)
Less than high school	18808 (13.0)	4416 (20.9)	812 (28.2)	2185 (25.6)	1361 (29.3)	369 (36.9)
Missing	1478 (1.0)	305 (1.4)	62 (2.1)	108 (1.3)	82 (1.8)	19 (1.9)
BMI, N (%)						
<25.0 Kg/m2	40923 (28.3)	4016 (19.0)	414 (14.4)	2505 (29.4)	1092 (23.5)	170 (17.0)
25-29.9 Kg/m2	74211 (51.4)	10262 (48.5)	1273 (44.1)	4059 (47.6)	2225 (47.9)	428 (42.8)
≥30.0 Kg/m2	29265 (20.3)	6876 (32.5)	1197 (41.5)	1967 (23.1)	1330 (28.6)	403 (40.3)
Urate, mean (SD), µmmol/L	351.83 (68.19)	364.19 (73.15)	368.70 (76.49)	352.27 (75.23)	360.01 (79.91)	371.86 (85.16)
Gout at baseline, N (%)	4571 (3.2)	1097 (5.2)	189 (6.6)	377 (4.4)	261 (5.6)	65 (6.5)

Note: Categorical data presented as numbers (percentages) and continuous data presented as mean (standard deviation). CMD, cardiometabolic disease; CMM, cardiometabolic multimorbidity; FCMD, first cardiometabolic disease; SD, standard deviation.

Table S5. Associations between serum uric acid, gout, FCMD, CMM, and mortality assessed using traditional Cox proportional hazards regression models.

Exposures	Outcomes	HR (95% CI)			P for interaction
		Overall	Female	Male	
SUA	FCMD	1.30 (1.28, 1.31)	1.45 (1.43, 1.47)	1.2 (1.18, 1.21)	P < 0.001
	CMM	1.43 (1.40, 1.47)	1.73 (1.66, 1.80)	1.26 (1.22, 1.31)	P < 0.001
	Death	1.09 (1.07, 1.10)	1.18 (1.16, 1.21)	1.03 (1.01, 1.05)	P < 0.001
Gout	FCMD	1.44 (1.37, 1.51)	2.05 (1.76, 2.38)	1.40 (1.33, 1.48)	P < 0.001
	CMM	1.70 (1.51, 1.92)	3.11 (2.19, 4.41)	1.62 (1.42, 1.84)	P < 0.001
	Death	1.19 (1.10, 1.27)	1.79 (1.45, 2.21)	1.13 (1.04, 1.22)	P < 0.001

Note: The model was adjusted for sex (male/female), age at recruitment (continuous), ethnic background (white British/non-white British), and the Townsend deprivation index (1st/2nd/3rd/4th/5th quintile). For sex-specific analyses, sex was not included in the model. CI, confidence interval; CMM, cardiometabolic multimorbidity; FCMD, first cardiometabolic disease; HR, hazard ratio; SUA, serum uric acid.

Table S6. Subgroup analyses of the associations between serum uric acid, gout, and multimorbidity in the cross-sectional study (N = 468,929).

Exposures	Subgroups	Cases	Total	OR (95% CI)	P for interaction
**SUA**	**Age**				< 0.001
	Below 60	45391	265792	1.311 (1.296, 1.327)	
	Above 60	60705	203137	1.282 (1.268, 1.296)	
	**Ethnic background**				0.162
	White British	93980	413981	1.265 (1.254, 1.276)	
	Non-white British	11567	52742	1.272 (1.242, 1.303)	
	**Townsend deprivation index**				0.121
	1st quintile	18815	93868	1.259 (1.235, 1.284)	
	2nd quintile	19678	93819	1.278 (1.254, 1.302)	
	3th quintile	20227	93682	1.258 (1.235, 1.281)	
	4th quintile	21590	93776	1.276 (1.254, 1.299)	
	5th quintile	25786	93784	1.264 (1.244, 1.284)	
**Gout**	**Age**				0.0327
	Below 60	1139	3892	2.271 (2.115, 2.437)	
	Above 60	2668	6053	1.916 (1.818, 2.019)	
	**Ethnic background**				0.733
	White British	3411	8933	1.946 (1.861, 2.035)	
	Non-white British	374	964	1.929 (1.682, 2.212)	

Exposures	Subgroups	Cases	Total	OR (95% CI)	P for interaction
	**Townsend deprivation index**				0.543
	1st quintile	619	1907	1.834 (1.659, 2.027)	
	2nd quintile	704	1918	2.048 (1.859, 2.257)	
	3th quintile	704	1940	1.936 (1.757, 2.132)	
	4th quintile	805	2066	1.964 (1.79, 2.155)	
	5th quintile	975	2114	1.957 (1.789, 2.139)	

Note: The model was adjusted for sex (male/female), age at recruitment (continuous), ethnic background (white British/non-white British), and the Townsend deprivation index (1st/2nd/3rd/4th/5th quintile). In stratified analyses, the stratifying covariate was excluded from the model. CI, confidence interval; OR, odds ratio; SUA, serum uric acid.

Table S7. Sensitivity analyses of associations between serum uric acid and multimorbidity in the cross-sectional study.

	Overall Cases/Total	OR (95% CI)	Female Cases/Total	OR (95% CI)	Male Cases/Total	OR (95% CI)	P for interaction
**Per SD increase in SUA** (removing outliers of SUA)	105102/466756	1.255 (1.245, 1.266)	58875/253910	1.403 (1.387, 1.419)	46227/212846	1.123 (1.11, 1.137)	< 0.001
**Per SD increase in SUA** (adjustment for potential covariates) #	106096/468929	1.257 (1.247, 1.267)	59089/254244	1.381 (1.365, 1.397)	47007/214685	1.153 (1.14, 1.167)	
**SUA quintiles**							< 0.001
Q1	18534/93742	1.00 (Reference)	9250/50844	1.00 (Reference)	9284/42898	1.00 (Reference)	
Q5	29508/93937	1.623 (1.588, 1.659)	17738/50924	1.958 (1.9, 2.017)	11770/43013	1.347 (1.305, 1.391)	
**HUA status**							< 0.001
Without HUA	86117/408188	1.00 (Reference)	49435/230371	1.00 (Reference)	36682/177817	1.00 (Reference)	
With HUA	19979/60741	1.723 (1.691, 1.757)	9654/23873	2.137 (2.078, 2.199)	10325/36868	1.465 (1.427, 1.504)	

Note: The model was primarily adjusted for sex (male/female), age at recruitment (continuous), ethnic background (white British/non-white British), and the Townsend deprivation index (1st/2nd/3rd/4th/5th quintile). For sex-specific analyses, sex was not included in the model. #, Potential covariates include drinking frequency, smoking status, physical activities, and diet quality scores. CI, confidence interval; HUA, hyperuricemia; OR, odds ratio; SD, standard deviation; SUA, serum uric acid.

Table S8. Sensitivity analyses of associations between gout, multimorbidity, and 36 chronic conditions in the cross-sectional study.

Chronic conditions	Overall Cases/Total	OR (95% CI)	Female Cases/Total	OR (95% CI)	Male Cases/Total	OR (95% CI)	P for interaction
Multimorbidity	3807/9945	1.731 (1.658, 1.807)	523/1022	2.27 (1.998, 2.58)	3284/8923	1.729 (1.651, 1.81)	< 0.001
Anemia	28/9945	2.576 (1.744, 3.806)	6/1022	2.689 (1.195, 6.052)	22/8923	2.105 (1.343, 3.3)	0.857
Angina	853/9945	1.469 (1.362, 1.584)	79/1022	2.087 (1.641, 2.654)	774/8923	1.467 (1.355, 1.587)	< 0.001
Anxiety/panic attacks	101/9945	0.984 (0.805, 1.202)	16/1022	1.054 (0.642, 1.73)	85/8923	0.993 (0.797, 1.238)	0.729
Asthma	1179/9945	1.087 (1.021, 1.157)	184/1022	1.466 (1.248, 1.722)	995/8923	1.074 (1.003, 1.15)	< 0.001
Atrial fibrillation	238/9945	1.692 (1.474, 1.941)	17/1022	1.908 (1.168, 3.12)	221/8923	1.733 (1.502, 2.001)	0.273
Bronchiectasis	30/9945	1.371 (0.946, 1.986)	7/1022	1.897 (0.896, 4.017)	23/8923	1.233 (0.804, 1.89)	0.401
Cancer	845/9945	1.061 (0.986, 1.141)	148/1022	1.2 (1.006, 1.431)	697/8923	1.021 (0.942, 1.107)	0.155
Chronic kidney disease	129/9945	4.238 (3.439, 5.224)	24/1022	6.004 (3.808, 9.467)	105/8923	4.273 (3.381, 5.399)	0.012
Chronic obstructive pulmonary disease	43/9945	0.883 (0.648, 1.202)	8/1022	1.46 (0.722, 2.954)	35/8923	0.803 (0.569, 1.131)	0.165
Chronic sinusitis	51/9945	0.902 (0.681, 1.195)	7/1022	0.951 (0.451, 2.006)	44/8923	0.9 (0.664, 1.221)	0.857
Cirrhosis	11/9945	1.234 (0.668, 2.282)	2/1022	1.813 (0.443, 7.41)	9/8923	1.292 (0.653, 2.554)	0.439
Dementia	0/9945	0 (0, 0)	0/1022	0 (0, 0)	0/8923	0 (0, 0)	1.000
Depression	487/9945	1.159 (1.055, 1.273)	80/1022	1.199 (0.953, 1.509)	407/8923	1.178 (1.063, 1.306)	0.626
Diabetes	1340/9945	1.894 (1.781, 2.014)	180/1022	3.171 (2.667, 3.771)	1160/8923	1.941 (1.817, 2.073)	< 0.001
Eczema or dermatitis	243/9945	1.028 (0.902, 1.172)	33/1022	1.405 (0.991, 1.992)	210/8923	0.98 (0.851, 1.128)	0.081
Epilepsy	52/9945	0.714 (0.542, 0.942)	6/1022	0.923 (0.413, 2.066)	46/8923	0.714 (0.532, 0.959)	0.449
Glaucoma	152/9945	1.02 (0.864, 1.203)	28/1022	2 (1.366, 2.928)	124/8923	0.931 (0.775, 1.118)	< 0.001
Heart failure	44/9945	3.065 (2.167, 4.337)	5/1022	3.025 (1.171, 7.813)	39/8923	3.285 (2.261, 4.774)	0.599
Hepatitis	32/9945	0.665 (0.467, 0.946)	7/1022	1.663 (0.787, 3.516)	25/8923	0.576 (0.386, 0.859)	0.024

Chronic conditions	Overall Cases/Total	OR (95% CI)	Female Cases/Total	OR (95% CI)	Male Cases/Total	OR (95% CI)	P for interaction
Hypertension	5514/9945	2.115 (2.026, 2.208)	591/1022	2.527 (2.205, 2.896)	4923/8923	2.177 (2.082, 2.277)	< 0.001
Inflammatory bowel disease	78/9945	0.94 (0.748, 1.181)	16/1022	1.777 (1.081, 2.923)	62/8923	0.855 (0.662, 1.105)	0.010
Irritable bowel syndrome	136/9945	0.948 (0.798, 1.127)	41/1022	1.299 (0.949, 1.778)	95/8923	0.886 (0.72, 1.09)	0.022
Meningitis	42/9945	0.978 (0.717, 1.334)	7/1022	1.738 (0.821, 3.675)	35/8923	0.911 (0.647, 1.282)	0.127
Migraine	115/9945	0.776 (0.644, 0.936)	28/1022	0.772 (0.53, 1.125)	87/8923	0.772 (0.622, 0.958)	0.968
Multiple sclerosis	17/9945	0.886 (0.547, 1.437)	7/1022	1.833 (0.868, 3.871)	10/8923	0.694 (0.369, 1.302)	0.036
Myocardial infarction	685/9945	1.4 (1.288, 1.521)	35/1022	1.982 (1.398, 2.81)	650/8923	1.403 (1.289, 1.528)	0.004
Osteoporosis	63/9945	0.826 (0.641, 1.064)	27/1022	0.786 (0.534, 1.156)	36/8923	0.911 (0.651, 1.275)	0.766
Parkinson’s disease	15/9945	0.589 (0.352, 0.985)	0/1022	0 (0, 0)	15/8923	0.628 (0.375, 1.052)	0.204
Peripheral vascular disease	21/9945	1.287 (0.828, 2)	5/1022	2.522 (1.039, 6.123)	16/8923	1.013 (0.61, 1.681)	0.197
Prostate problem	37/9945	0.964 (0.691, 1.345)	NA	NA	37/8923	0.964 (0.691, 1.345)	1.000
Rheumatoid arthritis	127/9945	1.366 (1.14, 1.637)	30/1022	1.641 (1.137, 2.367)	97/8923	1.299 (1.055, 1.601)	0.272
Schizophrenia	5/9945	0.329 (0.136, 0.798)	1/1022	1 (0.139, 7.22)	4/8923	0.311 (0.116, 0.835)	0.221
Stroke	321/9945	1.496 (1.33, 1.683)	34/1022	1.982 (1.397, 2.812)	287/8923	1.472 (1.299, 1.668)	0.058
Thyroid problem	386/9945	1.231 (1.107, 1.369)	151/1022	1.337 (1.122, 1.593)	235/8923	1.214 (1.061, 1.39)	0.197
Tuberculosis	70/9945	1.123 (0.881, 1.431)	10/1022	1.318 (0.703, 2.47)	60/8923	1.112 (0.854, 1.447)	0.554
Vestibular disorder	103/9945	1.024 (0.838, 1.25)	12/1022	1.298 (0.732, 2.302)	91/8923	0.993 (0.802, 1.229)	0.407
Multimorbidity #	3807/9945	1.954 (1.872, 2.039)	523/1022	2.593 (2.285, 2.944)	3284/8923	1.853 (1.77, 1.94)	< 0.001

Note: In the analyses multimorbidity and 36 chronic conditions, the model was adjusted for sex (male/female), age at recruitment (continuous), ethnic background (white British/non-white British), the Townsend deprivation index (1st/2nd/3rd/4th/5th quintile), and serum uric acid (continuous). #, The model was adjusted for sex (male/female), age at recruitment (continuous), ethnic background (white British/non-white British), the Townsend deprivation index (1st/2nd/3rd/4th/5th quintile), and potential covariates including drinking frequency, smoking status, physical activities, and diet quality scores. For sex-specific analyses, sex was not included in the model. CI, confidence interval; OR, odds ratio; SUA, serum uric acid.

Table S9. Sensitivity analyses of associations between serum uric acid and the trajectory of cardiometabolic multimorbidity (transition pattern A).

Exposure: SUA	Populations	Baseline → FCMD	FCMD → CMM	Baseline → Death	FCMD → Death	CMM → Death
Transforming SUA levels (continuous variable) into HUA (categorical variable)	Overall	1.618 (1.583, 1.653)	1.241 (1.172, 1.314)	1.168 (1.119, 1.219)	0.916 (0.865, 0.970)	1.191 (0.990, 1.432)
	Female	2.028 (1.959, 2.100)	1.382 (1.261, 1.515)	1.331 (1.246, 1.421)	0.867 (0.786, 0.956)	1.369 (1.013, 1.850)
	Male	1.417 (1.378, 1.457)	1.16 (1.078, 1.248)	1.078 (1.019, 1.140)	0.947 (0.882, 1.016)	1.097 (0.866, 1.389)
	P for interaction	< 0.001	0.004	< 0.001	0.112	0.214
Removing outliers of SUA	Overall	1.298 (1.284, 1.311)	1.122 (1.091, 1.153)	1.034 (1.014, 1.053)	0.912 (0.889, 0.936)	1.056 (0.965, 1.155)
	Female	1.46 (1.438, 1.483)	1.203 (1.153, 1.255)	1.125 (1.095, 1.156)	0.897 (0.860, 0.935)	1.121 (0.971, 1.293)
	Male	1.184 (1.168, 1.200)	1.066 (1.029, 1.106)	0.967 (0.942, 0.992)	0.925 (0.894, 0.956)	1.014 (0.903, 1.139)
	P for interaction	< 0.001	< 0.001	< 0.001	0.199	0.284
Additional adjustment for potential covariates #	Overall	1.303 (1.290, 1.316)	1.141 (1.111, 1.171)	1.047 (1.028, 1.066)	0.947 (0.924, 0.971)	1.048 (0.963, 1.142)
	Female	1.434 (1.413, 1.456)	1.209 (1.161, 1.260)	1.122 (1.093, 1.152)	0.918 (0.881, 0.956)	1.138 (0.990, 1.308)
	Male	1.209 (1.193, 1.224)	1.097 (1.060, 1.135)	0.995 (0.971, 1.020)	0.968 (0.938, 0.999)	0.989 (0.886, 1.104)
	P for interaction	< 0.001	0.001	< 0.001	0.041	0.162
Excluding participants who transitioned to different states on the same date	Overall	1.304 (1.292, 1.317)	1.128 (1.099, 1.158)	1.047 (1.028, 1.066)	0.944 (0.917, 0.971)	1.059 (0.969, 1.158)
	Female	1.46 (1.438, 1.482)	1.211 (1.162, 1.261)	1.135 (1.105, 1.165)	0.932 (0.891, 0.975)	1.165 (1.004, 1.352)

Exposure: SUA	Populations	Baseline → FCMD	FCMD → CMM	Baseline → Death	FCMD → Death	CMM → Death
	Male	1.2 (1.185, 1.216)	1.075 (1.039, 1.112)	0.985 (0.961, 1.010)	0.956 (0.921, 0.992)	1.003 (0.896, 1.123)
	P for interaction	< 0.001	< 0.001	< 0.001	0.314	0.120
Excluding events occurring within the first year of follow-up	Overall	1.299 (1.286, 1.312)	1.125 (1.095, 1.156)	1.046 (1.028, 1.066)	0.93 (0.907, 0.954)	1.078 (0.985, 1.179)
	Female	1.448 (1.426, 1.470)	1.202 (1.152, 1.254)	1.132 (1.103, 1.163)	0.915 (0.877, 0.953)	1.138 (0.980, 1.321)
	Male	1.199 (1.184, 1.215)	1.075 (1.038, 1.114)	0.986 (0.962, 1.011)	0.941 (0.911, 0.971)	1.039 (0.927, 1.165)
	P for interaction	< 0.001	< 0.001	< 0.001	0.244	0.343
Excluding events occurring within the first two years of follow-up	Overall	1.302 (1.289, 1.316)	1.13 (1.099, 1.163)	1.048 (1.029, 1.068)	0.923 (0.899, 0.947)	1.033 (0.937, 1.138)
	Female	1.446 (1.423, 1.469)	1.207 (1.155, 1.261)	1.134 (1.104, 1.166)	0.911 (0.873, 0.951)	1.028 (0.873, 1.210)
	Male	1.204 (1.188, 1.220)	1.081 (1.042, 1.122)	0.987 (0.962, 1.013)	0.931 (0.901, 0.963)	1.028 (0.910, 1.162)
	P for interaction	< 0.001	< 0.001	< 0.001	0.344	0.989
Excluding events occurring within the first three years of follow-up	Overall	1.303 (1.289, 1.317)	1.141 (1.106, 1.176)	1.049 (1.029, 1.070)	0.924 (0.899, 0.951)	1.029 (0.924, 1.145)
	Female	1.442 (1.419, 1.466)	1.228 (1.172, 1.287)	1.137 (1.105, 1.169)	0.908 (0.868, 0.949)	1.049 (0.881, 1.250)
	Male	1.206 (1.189, 1.223)	1.082 (1.040, 1.126)	0.986 (0.960, 1.013)	0.937 (0.904, 0.971)	1.008 (0.881, 1.155)
	P for interaction	< 0.001	< 0.001	< 0.001	0.227	0.730
Time intervals: 0.5 years	Overall	1.297 (1.285, 1.310)	1.128 (1.099, 1.158)	1.047 (1.028, 1.066)	0.935 (0.912, 0.958)	1.047 (0.960, 1.141)
	Female	1.453 (1.431, 1.474)	1.207 (1.159, 1.257)	1.134 (1.105, 1.165)	0.913 (0.877, 0.951)	1.127 (0.977, 1.299)
	Male	1.195 (1.180, 1.211)	1.077 (1.041, 1.114)	0.986 (0.962, 1.011)	0.95 (0.921, 0.981)	1.003 (0.900, 1.118)
	P for interaction	< 0.001	< 0.001	< 0.001	0.090	0.208
Time intervals: 1 year	Overall	1.297 (1.285, 1.310)	1.128 (1.100, 1.158)	1.047 (1.029, 1.066)	0.935 (0.912, 0.958)	1.05 (0.963, 1.144)

Exposure: SUA	Populations	Baseline → FCMD	FCMD → CMM	Baseline → Death	FCMD → Death	CMM → Death
	Female	1.453 (1.431, 1.475)	1.208 (1.160, 1.257)	1.134 (1.105, 1.164)	0.915 (0.879, 0.953)	1.127 (0.977, 1.299)
	Male	1.195 (1.180, 1.210)	1.077 (1.042, 1.114)	0.987 (0.963, 1.012)	0.949 (0.920, 0.979)	1.007 (0.903, 1.123)
	P for interaction	< 0.001	< 0.001	< 0.001	0.118	0.220

Note: The model was primarily adjusted for sex (male/female), age at recruitment (continuous), ethnic background (white British/non-white British), and the Townsend deprivation index (1st/2nd/3rd/4th/5th quintile). #, Potential covariates include drinking frequency, smoking status, physical activities, and diet quality scores. For sex-specific analyses, sex was not included in the model. CI, confidence interval; CMM, cardiometabolic multimorbidity; FCMD, first cardiometabolic disease; HR, hazard ratio; HUA, hyperuricemia; SUA, serum uric acid.

Table S10. Sensitivity analyses of associations between gout and the trajectory of cardiometabolic multimorbidity (transition pattern A).

Exposure: gout status	Populations	Baseline → FCMD	FCMD → CMM	Baseline → Death	FCMD → Death	CMM → Death
Excluding participants who transitioned to different states on the same date	Overall	1.448 (1.379, 1.520)	1.221 (1.082, 1.378)	1.122 (1.018, 1.236)	0.986 (0.860, 1.130)	0.687 (0.433, 1.090)
	Female	2.067 (1.775, 2.407)	1.732 (1.222, 2.456)	1.653 (1.258, 2.171)	1.227 (0.827, 1.821)	NA
	Male	1.41 (1.339, 1.485)	1.175 (1.033, 1.336)	1.059 (0.955, 1.174)	0.959 (0.829, 1.108)	0.752 (0.472, 1.197)
	P for interaction	< 0.001	0.046	0.010	0.305	NA
Excluding events occurring within the first year of follow-up	Overall	1.423 (1.354, 1.495)	1.225 (1.080, 1.389)	1.123 (1.018, 1.239)	0.984 (0.871, 1.111)	0.845 (0.554, 1.289)
	Female	1.994 (1.705, 2.332)	1.782 (1.235, 2.571)	1.659 (1.259, 2.185)	1.312 (0.909, 1.892)	NA
	Male	1.389 (1.319, 1.464)	1.176 (1.028, 1.344)	1.059 (0.953, 1.177)	0.954 (0.838, 1.084)	0.908 (0.592, 1.391)
	P for interaction	< 0.001	0.046	0.010	0.139	NA
Excluding events occurring within the first two years of follow-up	Overall	1.423 (1.352, 1.498)	1.189 (1.039, 1.360)	1.138 (1.029, 1.258)	0.956 (0.842, 1.084)	0.816 (0.486, 1.371)
	Female	1.981 (1.685, 2.329)	1.815 (1.241, 2.654)	1.637 (1.232, 2.175)	1.286 (0.880, 1.879)	NA
	Male	1.392 (1.319, 1.469)	1.132 (0.981, 1.307)	1.078 (0.968, 1.200)	0.925 (0.809, 1.057)	0.91 (0.540, 1.535)
	P for interaction	< 0.001	0.033	0.019	0.143	NA
Excluding events occurring within the first three years of follow-up	Overall	1.406 (1.332, 1.483)	1.179 (1.020, 1.363)	1.136 (1.023, 1.260)	0.939 (0.820, 1.075)	0.738 (0.414, 1.316)

Exposure: gout status	Populations	Baseline → FCMD	FCMD → CMM	Baseline → Death	FCMD → Death	CMM → Death
	Female	1.935 (1.633, 2.294)	1.778 (1.178, 2.684)	1.754 (1.320, 2.330)	1.181 (0.776, 1.797)	NA
	Male	1.377 (1.301, 1.457)	1.125 (0.964, 1.312)	1.065 (0.952, 1.191)	0.915 (0.794, 1.056)	0.804 (0.449, 1.440)
	P for interaction	< 0.001	0.055	0.005	0.306	NA
Additional adjustment for SUA levels (continuous variable)	Overall	1.225 (1.167, 1.286)	1.149 (1.018, 1.296)	1.096 (0.994, 1.208)	0.972 (0.863, 1.095)	0.72 (0.472, 1.099)
	Female	1.533 (1.318, 1.784)	1.433 (1.008, 2.037)	1.523 (1.159, 2.003)	1.228 (0.850, 1.773)	NA
	Male	1.265 (1.202, 1.331)	1.139 (1.001, 1.295)	1.068 (0.962, 1.185)	0.946 (0.834, 1.072)	0.802 (0.522, 1.230)
	P for interaction	< 0.001	0.098	0.011	0.290	NA
Additional adjustment for HUA (categorical variable)	Overall	1.258 (1.199, 1.320)	1.159 (1.027, 1.308)	1.077 (0.977, 1.187)	0.963 (0.855, 1.085)	0.711 (0.466, 1.085)
	Female	1.715 (1.475, 1.995)	1.532 (1.079, 2.175)	1.548 (1.178, 2.034)	1.19 (0.824, 1.717)	NA
	Male	1.276 (1.213, 1.343)	1.139 (1.001, 1.295)	1.039 (0.936, 1.153)	0.937 (0.827, 1.063)	0.79 (0.515, 1.212)
	P for interaction	< 0.001	0.071	0.009	0.338	NA
Additional adjustment for potential covariates	Overall	1.479 (1.410, 1.551)	1.252 (1.111, 1.412)	1.136 (1.031, 1.252)	0.966 (0.858, 1.087)	0.756 (0.495, 1.154)
	Female	1.947 (1.675, 2.264)	1.686 (1.188, 2.393)	1.578 (1.201, 2.074)	1.143 (0.792, 1.648)	NA
	Male	1.431 (1.360, 1.505)	1.209 (1.064, 1.374)	1.078 (0.972, 1.196)	0.952 (0.840, 1.079)	0.826 (0.539, 1.268)
	P for interaction	< 0.001	0.081	0.029	0.398	NA
Time intervals: 0.5 years	Overall	1.439 (1.372, 1.509)	1.218 (1.080, 1.374)	1.124 (1.020, 1.238)	0.948 (0.843, 1.068)	0.702 (0.453, 1.090)
	Female	2.052 (1.765, 2.386)	1.706 (1.203, 2.419)	1.648 (1.254, 2.165)	1.146 (0.794, 1.652)	NA
	Male	1.402 (1.333, 1.475)	1.174 (1.033, 1.334)	1.062 (0.958, 1.177)	0.93 (0.821, 1.054)	0.771 (0.495, 1.200)
	P for interaction	< 0.001	0.054	0.011	0.348	NA
Time intervals: 1 year	Overall	1.44 (1.373, 1.511)	1.221 (1.082, 1.377)	1.12 (1.016, 1.233)	0.956 (0.849, 1.076)	0.711 (0.458, 1.103)

Exposure: gout status	Populations	Baseline → FCMD	FCMD → CMM	Baseline → Death	FCMD → Death	CMM → Death
	Female	2.053 (1.766, 2.387)	1.707 (1.203, 2.420)	1.646 (1.253, 2.163)	1.152 (0.799, 1.661)	NA
	Male	1.404 (1.335, 1.476)	1.176 (1.035, 1.337)	1.058 (0.954, 1.172)	0.938 (0.827, 1.062)	0.782 (0.502, 1.217)
	P for interaction	< 0.001	0.054	0.010	0.348	NA

Note: The model was primarily adjusted for sex (male/female), age at recruitment (continuous), ethnic background (white British/non-white British), and the Townsend deprivation index (1st/2nd/3rd/4th/5th quintile). #, Potential covariates include drinking frequency, smoking status, physical activities, and diet quality scores. NA, The HR (95% CI) and P-value for interaction could not be reliably estimated due to an extremely low event count. For sex-specific analyses, sex was not included in the model. CI, confidence interval; CMM, cardiometabolic multimorbidity; FCMD, first cardiometabolic disease; HR, hazard ratio; HUA, hyperuricemia; SUA, serum uric acid.

Table S11. Details of the variables included in the study.

Variable	Field ID	Note	Instance	URL/Reference
**Exposures**				
Serum uric acid (SUA)	30880	Measured by uricase PAP analysis on a Beckman Coulter AU5800. Units of measurement are μmol/L. In the main analyses, SUA values were subsequently standardized to standard deviations for further analysis. In the sensitivity analyses, hyperuricemia (HUA) was defined as serum urate (field ID 30880) higher than 420 μmol/L among men or 360 μmol/L among women, according to previous research.	0	https://biobank.ctsu.ox.ac.uk/crystal/field.cgi?id=30880 doi: 10.3389/fnut.2022.920791 doi: 10.3389/fnut.2022.920791
Gout	131858	Date of the first occurrence of any code mapped to 3-character ICD10 M10. The code corresponds to "gout".	Singular	https://biobank.ctsu.ox.ac.uk/crystal/field.cgi?id=131858 doi: 10.1186/s12916-024-03836-8
**Outcomes**				
Chronic conditions	20002	Code for non-cancer illness. If the participant was uncertain of the type of illness they had had, then they described it to the interviewer (a trained nurse), who attempted to place it within the coding tree. If the illness could not be located in the coding tree, then the	0	doi: 10.1186/s12916-019-1339-0

Variable	Field ID	Note	Instance	URL/Reference
		interviewer entered a free-text description of it. These free-text descriptions were subsequently examined by a doctor and, where possible, matched to entries in the coding tree. Free-text descriptions that could not be matched with very high probability have been marked as "unclassifiable".		
Multimorbidity	NA	Multimorbidity was defined as the presence of two or more of 36 chronic conditions in participants	Singular	doi: 10.1186/s12916-019-1339-0
First cardiometabolic disease (FCMD),	NA	FCMD was defined as the first occurrence of any of the following three cardiometabolic diseases (type 2 diabetes, ischemic heart disease, and stroke).	Singular	doi: 10.1093/eurheartj/ehab413<br>doi: 10.1016/j.ebiom.2022.104282
Cardiometabolic multimorbidity (CMM)	NA	CMM was defined as the co-occurrence of at least two of the cardiometabolic diseases (type 2 diabetes, ischemic heart disease, and stroke).	Singular	doi: 10.1093/eurheartj/ehab413<br>doi: 10.1016/j.ebiom.2022.104282
Cardiometabolic diseases				
(1) Type 2 diabetes	Category 1712	ICD codes: E11 and E14	Singular	doi: 10.1093/eurheartj/ehab413<br>doi: 10.1016/j.ebiom.2022.104282
(2) Ischemic heart	Catego	ICD codes: I20-I25	Singular	doi: 10.1093/eurheartj/ehab413

Variable	Field ID	Note	Instance	URL/Reference
disease	ry 1712			doi: 10.1016/j.ebiom.2022.104282
(3) Stroke	Category 1712	ICD codes: I61 and I63	Singular	doi: 10.1093/eurheartj/ehab413<br>doi: 10.1016/j.ebiom.2022.104282
Loss to follow up	191	Date lost to follow-up	Singular	https://biobank.ndph.ox.ac.uk/showcase/field.cgi?id=191
Death	40000	Date of death linkage to death registries.	Singular	https://biobank.ndph.ox.ac.uk/showcase/refer.cgi?id=115559<br>doi: 10.1016/j.mayocp.2021.02.011
**Covariates (main analyses)**				
Sex	31	Mostly acquired from the central registry at recruitment.	Singular	https://biobank.ctsu.ox.ac.uk/crystal/field.cgi?id=31
Age	21022	This is a derived variable based on date of birth and date of attending an initial assessment center and refers to the age of the participant on the day they attended an Initial Assessment Centre, truncated to whole year.	Singular	https://biobank.ctsu.ox.ac.uk/crystal/field.cgi?id=21022

Variable	Field ID	Note	Instance	URL/Reference
Ethnic background	21000	Ethnic background was categorized as white British and non-white British, according to a previous study.	0	https://biobank.ndph.ox.ac.uk/showcase/field.cgi?id=21000<br>10.1016/j.bbi.2018.11.007
Townsend deprivation index	22189	Townsend Deprivation Index, a measure of socioeconomic deprivation, was divided into quintiles, with the first quintile representing the least deprived and the fifth quintile the most deprived.	Singular	https://biobank.ndph.ox.ac.uk/showcase/field.cgi?id=22189<br>doi: 10.1186/s12916-022-02544-5
**Covariates (sensitivity analyses)**				
Smoking status	20116	Smoking status was defined as current, past, and never, according to a previous study.	0	https://biobank.ctsu.ox.ac.uk/crystal/field.cgi?id=20116<br>doi: 10.2337/dc21-1512
Drinking frequency	1558	(1) Touchscreen question: "About how often do you drink alcohol?"<br>(2) Answers: 1: Daily or almost daily; 2: Three or four times a week; 3: Once or twice a week; 4: One to three times a month; 5: Special occasions only.<br>(3) Alcohol intake frequency was divided into three levels (frequent, infrequent, and never), according to previous research.	0	https://biobank.ctsu.ox.ac.uk/crystal/field.cgi?id=1558<br>doi: 10.1053/j.ajkd.2020.11.018

Variable	Field ID	Note	Instance	URL/Reference
Physical activity	22040	Total Metabolic Equivalent Task (MET) minutes per week for all activities, including walking, moderate, and vigorous activity. Physical activity was categorized into three levels based on MET: low (< 600), moderate (600-3000), and high (> 3000), according to previous research.	0	https://biobank.ndph.ox.ac.uk/showcase/field.cgi?id=22040<br>doi:10.1093/eurheartj/ehy695
Diet quality scores	NA	The diet quality scores were constructed based on the intake of four types of food, including fruits and vegetables (i.e., fresh fruit, dried fruit, cooked vegetables, salad/raw vegetables), oily fish, red meat (i.e., beef, lamb/mutton, pork), and processed meat. The scores were divided into five levels: 0, 1, 2, 3, and 4, according to previous research.	0	doi: 10.1016/S2468-2667(18)30200-7
(1) Fresh fruit intake	1309	(1) Touchscreen question: "About how many pieces of FRESH fruit would you eat per DAY? (Count one apple, one banana, 10 grapes, etc., as one piece; put '0' if you do not eat any)"<br>(2) Answers: The answer could only contain integer values.	0	https://biobank.ctsu.ox.ac.uk/crystal/field.cgi?id=1309
(2) Dried fruit	26092	Total weight of food group: Dried fruit. Estimated	0	https://biobank.ctsu.ox.ac.uk/crystal/field.cgi?id=26092

Variable	Field ID	Note	Instance	URL/Reference
		intake from the past 24 hours.		
(3) Cooked vegetable intake	1289	(1) Touchscreen question: "On average, how many heaped tablespoons of COOKED vegetables would you eat per DAY? (Do not include potatoes; put '0' if you do not eat any)"<br>(2) Answers: The answer could only contain integer values.	0	https://biobank.ctsu.ox.ac.uk/crystal/field.cgi?id=1289
(4) Salad/raw vegetable intake	1299	(1) Touchscreen question: "On average, how many heaped tablespoons of SALAD or RAW vegetables would you eat per DAY? (Include lettuce, tomato in sandwiches; put '0' if you do not eat any)"<br>(2) Answers: The answer could only contain integer values.	0	https://biobank.ctsu.ox.ac.uk/crystal/field.cgi?id=1299
(5) Oily fish intake	1329	(1) Touchscreen question: "How often do you eat oily fish? (e.g., sardines, salmon, mackerel, herring)"<br>(2) Answers: 0: Never 1: Less than once a week 2: Once a week 3: 2-4 times a week 4: 5-6 times a week 5: Once or more daily	0	https://biobank.ctsu.ox.ac.uk/crystal/field.cgi?id=1329
(6) Beef intake	1369	(1) Touchscreen question: "How often do you eat beef? (Do not count processed meats)"	0	https://biobank.ctsu.ox.ac.uk/crystal/field.cgi?id=1369

Variable	Field ID	Note	Instance	URL/Reference
		(2) Answers: 0: Never 1: Less than once a week 2: Once a week 3: 2-4 times a week 4: 5-6 times a week 5: Once or more daily		
(7) Lamb/mutton intake	1379	(1) Touchscreen question: "How often do you eat lamb/mutton? (Do not count processed meats)"<br>(2) Answers: 0: Never 1: Less than once a week 2: Once a week 3: 2-4 times a week 4: 5-6 times a week 5: Once or more daily	0	https://biobank.ctsu.ox.ac.uk/crystal/field.cgi?id=1379
(8) Pork intake	1389	(1) Touchscreen question: "How often do you eat pork? (Do not count processed meats such as bacon or ham)"<br>(2) Answers: 0: Never 1: Less than once a week 2: Once a week 3: 2-4 times a week 4: 5-6 times a week 5: Once or more daily	0	https://biobank.ctsu.ox.ac.uk/crystal/field.cgi?id=1389
(9) Processed meat intake	1349	(1) Touchscreen question: "How often do you eat processed meats (such as bacon, ham, sausages, meat pies, kebabs, burgers, chicken nuggets)?"<br>(2) Answers: 0: Never 1: Less than once a week 2: Once a week 3: 2-4 times a week 4: 5-6 times a week 5: Once or more daily	0	https://biobank.ctsu.ox.ac.uk/crystal/field.cgi?id=1349

Table S12. 36 chronic conditions included in the cross-sectional study.

Code	Chronic conditions
1	Anemia
2	Angina
3	Anxiety/panic attacks
4	Asthma
5	Atrial fibrillation
6	Bronchiectasis
7	Cancer
8	Chronic kidney disease
9	Chronic obstructive pulmonary disease
10	Chronic sinusitis
11	Cirrhosis
12	Dementia
13	Depression
14	Diabetes
15	Eczema or dermatitis
16	Epilepsy
17	Glaucoma
18	Heart failure
19	Hepatitis
20	Hypertension

Code	Chronic conditions
21	Inflammatory bowel disease
22	Irritable bowel syndrome
23	Meningitis
24	Migraine
25	Multiple sclerosis
26	Myocardial infarction
27	Osteoporosis
28	Parkinson’s disease
29	Peripheral vascular disease
30	Prostate problem
31	Rheumatoid arthritis
32	Schizophrenia
33	Stroke
34	Thyroid problem
35	Tuberculosis
36	Vestibular disorder

Supplementary Document

STROBE Statement—checklist of items that should be included in reports of observational studies.

	Item No	Recommendation	Page No
**Title and abstract**	1	(*a*) Indicate the study's design with a commonly used term in the title or the abstract	1
		(*b*) Provide in the abstract an informative and balanced summary of what was done and what was found	3
**Introduction**			
Background/rationale	2	Explain the scientific background and rationale for the investigation being reported	4-5
Objectives	3	State specific objectives, including any prespecified hypotheses	5
**Methods**			
Study design	4	Present key elements of study design early in the paper	19
Setting	5	Describe the setting, locations, and relevant dates, including periods of recruitment, exposure, follow-up, and data collection	18-19
Participants	6	(*a*) *Cohort study*—Give the eligibility criteria, and the sources and methods of selection of participants. Describe methods of follow-up<br>*Case-control study*—Give the eligibility criteria, and the sources and methods of case ascertainment and control selection. Give the rationale for the choice of cases and controls<br>*Cross-sectional study*—Give the eligibility criteria, and the sources and methods	19, Figure. S7

		of selection of participants	
		(*b*) *Cohort study*—For matched studies, give matching criteria and number of exposed and unexposed<br>*Case-control study*—For matched studies, give matching criteria and the number of controls per case	NA
Variables	7	Clearly define all outcomes, exposures, predictors, potential confounders, and effect modifiers. Give diagnostic criteria, if applicable	19-20
Data sources/ measurement	8*	For each variable of interest, give sources of data and details of methods of assessment (measurement). Describe comparability of assessment methods if there is more than one group	19-20, Table S11
Bias	9	Describe any efforts to address potential sources of bias	22-23
Study size	10	Explain how the study size was arrived at	NA
Quantitative variables	11	Explain how quantitative variables were handled in the analyses. If applicable, describe which groupings were chosen and why	20, Table S11
Statistical methods	12	(*a*) Describe all statistical methods, including those used to control for confounding	20-22
		(*b*) Describe any methods used to examine subgroups and interactions	22
		(*c*) Explain how missing data were addressed	20
		(*d*) *Cohort study*—If applicable, explain how loss to follow-up was addressed<br>*Case-control study*—If applicable, explain how matching of cases and controls was addressed<br>*Cross-sectional study*—If applicable, describe analytical methods taking account of sampling strategy	21
		(*e*) Describe any sensitivity analyses	22-23

Continued on next page

**Results**			**Page No**
Participants	13*	(a) Report numbers of individuals at each stage of study—eg numbers potentially eligible, examined for eligibility, confirmed eligible, included in the study, completing follow-up, and analysed	Figure. S7, 5-7, 8-9
		(b) Give reasons for non-participation at each stage	Figure. S7, 5-7, 8-9
		(c) Consider use of a flow diagram	Figure. S7, Figs. 2-3
Descriptive data	14*	(a) Give characteristics of study participants (eg demographic, clinical, social) and information on exposures and potential confounders	5-6, 8
		(b) Indicate number of participants with missing data for each variable of interest	NA
		(c) *Cohort study*—Summarise follow-up time (eg, average and total amount)	8
Outcome data	15*	*Cohort study*—Report numbers of outcome events or summary measures over time	8-9
		Case-control study—Report numbers in each exposure category, or summary measures of exposure	NA
		Cross-sectional study—Report numbers of outcome events or summary measures	5-6
Main results	16	(*a*) Give unadjusted estimates and, if applicable, confounder-adjusted estimates and their precision (eg, 95% confidence interval). Make clear which confounders were adjusted for and why they were included	5-11
		(*b*) Report category boundaries when continuous variables were categorized	Table

			S11
		(*c*) If relevant, consider translating estimates of relative risk into absolute risk for a meaningful time period	NA
Other analyses	17	Report other analyses done—eg analyses of subgroups and interactions, and sensitivity analyses	10-11
**Discussion**			
Key results	18	Summarise key results with reference to study objectives	11
Limitations	19	Discuss limitations of the study, taking into account sources of potential bias or imprecision. Discuss both direction and magnitude of any potential bias	17-18
Interpretation	20	Give a cautious overall interpretation of results considering objectives, limitations, multiplicity of analyses, results from similar studies, and other relevant evidence	18
Generalisability	21	Discuss the generalisability (external validity) of the study results	18
**Other information**			
Funding	22	Give the source of funding and the role of the funders for the present study and, if applicable, for the original study on which the present article is based	23-24

*Give information separately for cases and controls in case-control studies and, if applicable, for exposed and unexposed groups in cohort and cross-sectional studies.

Note: An Explanation and Elaboration article discusses each checklist item and gives methodological background and published examples of transparent reporting. The STROBE checklist is best used in conjunction with this article (freely available on the Web sites of PLoS Medicine at http://www.plosmedicine.org/, Annals of Internal Medicine at http://www.annals.org/, and Epidemiology at http://www.epidem.com/). Information on the STROBE Initiative is available at www.strobe-statement.org.
